# Effects of isolation and confinement on gastrointestinal microbiota–a systematic review

**DOI:** 10.3389/fnut.2023.1214016

**Published:** 2023-07-10

**Authors:** Bea Klos, Christina Steinbach, Jasmin Ketel, Claude Lambert, John Penders, Joël Doré, Paul Enck, Isabelle Mack

**Affiliations:** ^1^Department of Psychosomatic Medicine and Psychotherapy, University Hospital Tübingen, Tübingen, Germany; ^2^CIRI–Immunology Lab University Hospital, Saint-Étienne, France; ^3^LCOMS/ENOSIS Université de Lorraine, Metz, France; ^4^Department of Medical Microbiology, Infectious Diseases and Infection Prevention, Maastricht University Medical Center, CAPHRI Care and Public Health Research Institute, Maastricht, Netherlands; ^5^Department of Medical Microbiology, Infectious Diseases and Infection Prevention, Maastricht University Medical Center, School of Nutrition and Translational Research in Metabolism, Maastricht, Netherlands; ^6^UMR Micalis Institut, INRA, Paris-Saclay University, Jouy-En-Josas, France

**Keywords:** isolation, confinement, human, microbiota, gut, gastrointestinal, space, Antarctica

## Abstract

**Purpose:**

The gastrointestinal (GI) microbiota is a complex and dynamic ecosystem whose composition and function are influenced by many internal and external factors. Overall, the individual GI microbiota composition appears to be rather stable but can be influenced by extreme shifts in environmental exposures. To date, there is no systematic literature review that examines the effects of extreme environmental conditions, such as strict isolation and confinement, on the GI microbiota.

**Methods:**

We conducted a systematic review to examine the effects of isolated and confined environments on the human GI microbiota. The literature search was conducted according to PRISMA criteria using PubMed, Web of Science and Cochrane Library. Relevant studies were identified based on exposure to isolated and confined environments, generally being also antigen-limited, for a minimum of 28 days and classified according to the microbiota analysis method (cultivation- or molecular based approaches) and the isolation habitat (space, space- or microgravity simulation such as MARS-500 or natural isolation such as Antarctica). Microbial shifts in abundance, alpha diversity and community structure in response to isolation were assessed.

**Results:**

Regardless of the study habitat, inconsistent shifts in abundance of 40 different genera, mainly in the phylum Bacillota (formerly Firmicutes) were reported. Overall, the heterogeneity of studies was high. Reducing heterogeneity was neither possible by differentiating the microbiota analysis methods nor by subgrouping according to the isolation habitat. Alpha diversity evolved non-specifically, whereas the microbial community structure remained dissimilar despite partial convergence. The GI ecosystem returned to baseline levels following exposure, showing resilience irrespective of the experiment length.

**Conclusion:**

An isolated and confined environment has a considerable impact on the GI microbiota composition in terms of diversity and relative abundances of dominant taxa. However, due to a limited number of studies with rather small sample sizes, it is important to approach an in-depth conclusion with caution, and results should be considered as a preliminary trend. The risk of dysbiosis and associated diseases should be considered when planning future projects in extreme environments.

**Systematic review registration:**

https://www.crd.york.ac.uk/prospero/, identifier CRD42022357589.

## 1. Introduction

The indigenous microbiota of the human is known to be closely associated with various physiological functions, with the gastrointestinal (GI) microbiota in particular playing a prominent role in the protection against infection (1, 2), in the digestion of food (3), or in the production of neurotransmitters (4). There is also evidence that intestinal microbes affect energy metabolism (5), intestinal epithelial proliferation (6) and immune response in the host (1). As a consequence, there is increasing evidence of a link between GI dysbiosis and the development of metabolic (obesity), infectious and immune-mediated diseases (allergies, inflammatory bowel diseases) (7).

There are significant interindividual variations in GI microbiota composition, with each individual harboring a unique combination of microbial species (8, 9). The microbiota richness, diversity and community structure are quantified using various ecological measures and indices. Alpha diversity refers to both the number of different species and their distribution (evenness) within a given microbial habitat, and is therefore divided into richness and biodiversity (10). Beta-diversity compares the structure of microbial communities and is determined by the degree of similarity/dissimilarity between communities within different microbial habitats (11).

Generally, the microbiota composition is dependent of environment and food intake in early childhood but is considered to be rather stable in adulthood (12, 13). However, extreme hin diet (14, 15), bariatric surgery (16, 17), the use of medications (especially antibiotics) (18–20) and high hygiene (21) can significantly affect the composition and the function of the indigenous microbiome, and could contribute considerably to a dysbiosis between beneficial and potentially harmful bacteria. Besides environmental factors, genetic background (22, 23) and local immunity (24) also have an important impact on the composition of the microbial community; however, studies suggest that external factors may have a greater impact on dysbiosis than genetic factors (22, 25).

Current literature supports changes in intestinal microbiota due to extreme environmental conditions such as extreme temperatures (26, 27), high altitudes (28–30) or radiation (31–33). Studies in mice have demonstrated that the gut microbiota changes significantly during both real spaceflights and simulated microgravity (34, 35). Additional changes in the microbiota occurred during a real spaceflight that were beyond those observed in ground-controlled animals. This suggests that the space experience has unique features that cause changes in the microbiome (36, 37).

If environmental bacterial load influences the gut microbiota, what happens in case of poor bacterial diversity exposure? The effects of antigen-limited or poor environments on the human gut microbiota have to date received rather little attention. There is evidence that spaceflights can cause dysbiosis in humans, with a reduction in symbiotic microbes and a rise in opportunistic pathogens, affecting both microbial diversity and community structure significantly (25, 37). Several space mission experiments have detected changes in GI bacterial species composition and function (25, 38–40), bacterial gene expression and protein regulation (41) suggesting a potential host-microbial interaction that may contribute to a decline in protein metabolism in the host (35) during spaceflight and after its completion, but results remain conflicting (42). Another long-term spaceflight found neither a reduction in richness nor a change in community structure in the in-flight samples compared to the pre-flight and post-flight samples. The highly variable core microbiome is expected to be present in both spaceflight astronauts and ground-based controls with fecal microbial communities differing significantly between spaceflight astronauts and ground-based controls and remaining distinct over the time (25). Along with the host immune system, the core microbiome is thought to be pivotal in the maintenance of human health even during and after spaceflights (39).

Terrestrial ways to limit antigen exposure include exposure to environmental conditions that are highly challenging for humans, such as in Antarctica, or in specialized facilities known as space simulation units. These units simulate the conditions of spaceflights, including weightlessness, increased radiation, and other factors. The stay of a healthy subject in an environment with altered parameters is accompanied by dysbiotic changes resulting in a decrease in colonization resistance of the intestine and integumentary tissues (43). Within the MARS-500 experiment (44), stool samples were collected and analyzed during a long-term stay (520 days) in a facility simulating spaceflight. Data showed significant changes in the taxonomic composition during the initial stages of the experiment, but the basic composition of the intestinal ecosystem remained unchanged in all 5 individuals without changes in the enterotypes of individual taxonomic groups. After the confinement the taxa tended to reverse to their original state. By clustering the gut microbiota of subjects into two enterotypes, Chen et al. (45) suggested that the composition of the gut microbiota is a crucial factor in the adaptability of individuals to antigen-limited environment exposure, as subjects showed either no significant differences in health indicators before and after confinement or experienced several health problems after confinement, such as increased uric acid, anxiety, and constipation, and lack of sleep. Similarly, another simulation experiment (46) reported mixed results: The data showed increased abundances of the genera *Roseburia*, *Prevotella*, *Lachnospira*, and *Phascolarctobacterium*, while abundances of the genera *Faecalibacterium*, *Parabacteroides*, *Bacteroides*, *Bifidobacterium*, and *Anaerostipes* dropped. However, it remains unclear whether these effects result from exposure to an antigen-limited or antigen-poor environment or from microgravity, which has already been linked to changes in virulence factors, bacterial stress responses and biofilm formation (47).

Although there have been various studies on the changes or stability of microbiota in response to antigen-limited or poor environments, the underlying mechanisms behind these observations are not yet fully understood and remain an active area of scientific inquiry. Effect of exposure is time dependent and long-term experiments on a sufficient number of volunteers are rare. A systematic investigation of the impact of a long-term residence (>28 days) in such an environment on the composition, diversity and stability of the GI microbiota has not yet been conducted. Therefore, we aim to fill this gap in knowledge through a comprehensive review of the available literature and a systematic search for relevant studies. We ask for two questions:

1. What is the effect of long-term residence (>28 days) in an antigen-limited or poor environment on the relative abundances of key bacterial taxa, and what happens to GI microbiota in terms of richness and biodiversity (alpha diversity) and community structure?
2. How reversible are the isolation-induced effects on the relative abundances, alpha diversity and community structure after the exposure to an antigen-limited or poor environment?

## 2. Materials and methods

### 2.1. Literature information sources and search strategy

This review was developed and executed according to the Preferred Reporting Items for Systematic Reviews and Meta-Analyses (PRISMA) guidelines (48). To identify all relevant studies examining the effect of isolation and confinement on the human gut microbiota the databases PubMed, Web of Science, Cochrane Library (Wiley) and EBM-Reviews (Ovid) Cochrane Library were searched on September 2^nd^ 2022. The protocol of this systematic review is registered on the PROSPERO platform with the registration number CRD42022357589. The full search strategy was conducted in assistance with a specialized librarian and is documented in the supporting information (Supplementary Text S1). It consists of three models: isolation condition, human gut microbiota and exclusion of animals. For the search, a very specific search term was chosen to represent the isolation conditions as best as possible. Broader search terms were also tested, but not all relevant studies were found.

### 2.2. Eligibility criteria

Inclusion criteria were established based on the five PICOS dimensions, i.e., participants, interventions, comparator, outcome and study design (49).

Participants: Healthy adults, regardless of sex, age, or weight status, who had been under isolation conditions for at least 28 days were included.

Intervention: Isolation in an environment with constant / reduced antigen exposure. Such isolation conditions are found (a) in space missions; (b) in isolation simulations, such as MARS-500, SIRIUS and Lunar Palace-1 or in bed-rest studies; (c) in extreme environmental conditions, such as in Antarctica, Arctic and Siberia. The intake of probiotics was allowed. Studies that focused on the use of antibiotics for preventing infectious and inflammatory diseases in humans were excluded.

Comparator: Studies with or without control groups met eligibility criteria.

Outcome: Assessment of the microbiota of the human GI tract.

Study design: Randomized controlled trials or non-randomized controlled trials with any publication date and written in English, German and Russian. Only original articles were included.

### 2.3. Study selection and organization

To identify eligible studies, the search results of the databases were combined, and the duplicates were removed. Two authors (BK and CS) independently screened titles and abstracts to identify relevant trials. Full-text articles were evaluated regarding their eligibility (BK, CS), with uncertainties being discussed between the authors (<3% cases). A third author (IM) was involved if the discrepancy persisted.

Throughout the decades, the methods of microbial analysis changed from cultivation and cell counting to molecular-based approaches like next-generation sequencing, micro-arrays or quantitative polymerase chain reaction. Due to the huge diversity between the method procedures and the associated heterogeneity of the outcomes, the results were assessed separately from each other. The studies were classified into two groups according to microbiota analysis method:

Group 1–Cultivation-based approaches for microbiota analysis.

Group 2–Molecular-based approaches for microbiota analysis.

Additionally, subgroups were created to provide a more homogenous summary of findings.

Subgroup 1–Isolation caused by space missions.

Subgroup 2–Isolation caused by spaceflight- or gravity simulators, e.g., MARS-500, bioregenerative life support systems (BLSS) or bed rest-studies.

Subgroup 3–Isolation in a natural, earth-bound habitat, e.g., Antarctica.

Certain experiments may have resulted in multiple publications concerning the GI microbiota. As the outcomes may differ in detail and description, all publications are listed in the tables. However, a summary of these studies is provided in the text and data evaluation sections.

### 2.4. Data items and statistics

The following information was extracted from each included article for groups 1 and 2: study characteristics, conditions of isolation, methods of GI microbiota analysis and outcomes. Each study’s characteristics are reported using the original data and summarized in tabular form. Characteristics across the studies are presented as mean, minimum and maximum for sample size, age, body mass index (BMI) and study length.

Primary outcomes concerning the GI microbiota were alpha diversity (richness and biodiversity), community structure/beta diversity and significant shifts in the abundance of individual microbial taxa (according to the current version of the International Code of Nomenclature of Prokaryotes (50)). Significant differences for alpha diversity, community structure, and taxonomy abundances at both phylum and genus level were summarized for isolation/in-mission and post-isolation/post-mission. We also described conclusions on a pre/post comparison if this was possible. For the microbial abundance outcomes in studies using molecular based methods which were not specifically referred to in text, no effect was presumed and written as unchanged (↔). If data was only presented graphically, the abundance shifts were extracted as best as conceivable. The graphical representation of relative abundance shifts was limited to the phylum level to avoid over- or underreporting of data. Non-isolated control subjects were not considered in further analysis. The data were analyzed separately for groups and subgroups. Finally, data was summarized across the habitats. Secondary outcomes including anthropometric, clinical, behavioral, psychological changes were also retrieved.

### 2.5. Risk of bias

For the included studies, a risk of bias assessment was performed using Risk of Bias In Non-randomized Studies of Interventions (ROBINS-I) tool (51). As only non-randomized isolation interventions were included in this systematic review, we chose the Cochrane tool as this tool views each study as an attempt to emulate a hypothetical pragmatic randomized trial and covers seven distinct domains through which bias might be introduced. In the first two domains, issues related to confounding and selection of participants are addressed before the interventions to be compared (“baseline”), while the third domain discusses intervention classification. In the remaining four domains, the following issues are addressed after the start of interventions: biases due to deviations from intended interventions, missing data, measurement of outcomes, and selection of the reported result.

The rating ranged between “Low risk,” “Moderate risk,” “Serious risk” and “Critical risk” of bias. The authors declare that “Low risk” corresponds to the risk of bias in a high-quality trial, however, due to the limited number of studies found, no study was excluded for risk of bias.

## 3. Results

**Figure 1:**
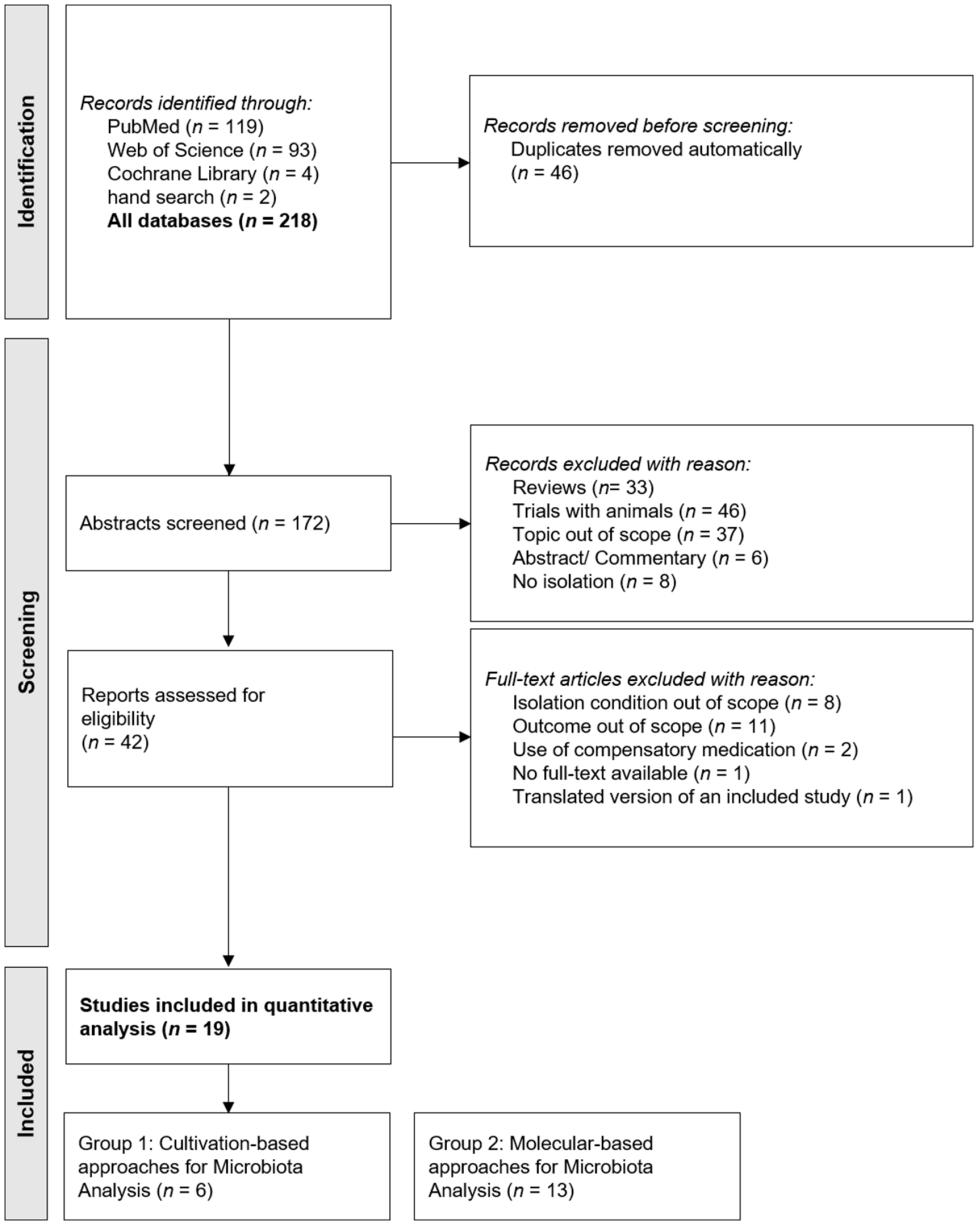
PRISMA flow chart for study inclusion.

The literature search process for identification of eligible studies is shown in Figure 1. Out of 218 identified studies, 19 studies remained for qualitative analysis. Six articles were categorized in group 1 (cultivation-based approaches for microbiota analysis) and 13 articles in group 2 (molecular-based approaches for microbiota analysis).

### 3.1. Summary of the study characteristics

A detailed overview of the characteristics for the single trials is presented in Table 1 for cultivation methods and Table 2 for molecular-based approaches. The characteristics across the studies are summarized in the text.

**Table 1:** Characteristics of studies using cultivation methods, characteristics are split according to corresponding isolation habitats.

Study ID	Author (year)	Origin	Isolation	Length (days)	Sample size (participants)	Age (years)	Sex, % f	Pre-BMI (kg/m^2^)	Sampling time	Cultured microbes	Data derivation	Diet	Additional intake
Subgroup 1: Isolation caused by space missions													
1	Lizko et al. (52)	USSR	Salute 4^1^ Sojus 17	30	2	43	0	N.R.	Pr/Du/Po	*Staphylococcus*, *Streptococcus*, *Proteus*, *E. coli*, *Lactobacillu*s, Bacteroids, Bifidobacteria, Spore-forming bacteria, Yeast	Text	N.R.	No
			Salute 4^1^ Sojus 18	63	2	32, 40							Yes (not further described)
Subgroup 2: Isolation caused by spaceflight- or gravity simulators													
2	Chen et al. (53)	CHN	6 ° HDBR^2^	45	7	26.13 ± 4.05	0	21.7	Pr/Du/Po	N.R.	Text	Controlled, but no special diet	N.R.
3	Cordaro et al. (54)	USA	Oxygen chamber^3^	56	4	27–29	0	23.9	Pr/Du/Po	Coliforms, *Proteus*, Salmonellae, Staphylococci, Enterococci, *Streptococcus salivarius*, Yeasts, Diphtheroids, Bacteroids, Clostridia, Lactobacilli	Graphic	Compressed freeze-dried cubes	N.R.
4	Gall and Riely (55)	USA	Experimental activity facility	45	4	21–34	0	N.R.	Du	Staphylococci, Streptococci, Lactobacilii, *Haemophilus*, Neisseria, Enterobacteriaceae, *Shigella, Salmonella, E. coli*, *Klebsiella*, Yeast, *Proteus, Pseudomonas*	Text	Space-type freeze dehydrated diet (60 foods) and equivalent fresh food	N.R.
5	Rerberg et al. (56)	SIB	BIOS-3^4^	120–180	4	N.R.	N.R.	N.R.	Du	N.R.	Text	N.R.	N.R.
6	Shilov et al. (57)	RUS	Hermetic chamber	365	3	N.R.	N.R.	N.R.	Pr/Du/Po	Bifidobacteria, Lactobacilli, *E. coli,* Streptococci, Clostridia, Staphylococci, Yeast, Protea	Text + graphic	Common mixed diet from natural products	N.R.

“Isolation”: Isolated and confined extreme environments. “Length”: length of the isolation intervention, no pre/post surveys are considered. The sampling times are either before the start of the intervention (“Pre”), during the isolation itself (“During”), after the isolation (“Post”), or a combination of more than one of these times. “Additional intake”: intake of pro/pre/antibiotics during the isolation period.

^1^A Soviet space station. ^2^Gravity simulation. ^3^Oxygen chamber has an oxygen content of 70%, a helium content of 30%. ^4^Experimental project of a closed ecosystem. 6°HDBR, −6° head-down bed rest; CHN, China; Du, During; f, female; N.R., not reported; Po, Post; Pr, Pre; RUS, Russia; SIB, Siberia; USA, United States of America; USSR, Union of Soviet Socialist Republics; BMI, Body Mass Index.

**Table 2:** Study characteristics of studies using molecular-based approaches, characteristics are split according to corresponding isolation habitats.

Study ID	Author (Year)	Origin	Isolation	Length (days)	Sample Size	Age (years)	Sex, % f.	Pre-BMI (kg/m^2^)	Sampling point	Analysis method	Microbiota classification	Data derivation	Diet	Additional intake
Subgroup 1: Isolation caused by space missions														
1	Garret-Bakelman et al. (25)	USA	Spaceflight	340	1	50	0	N.R.	Pr/Du/Po	WMGS (Illumina)	N.R.	Text + graphic	Restricted diet	N.R.
2	Liu et al. (40)	CHN	Spaceflight	35	2	N.R.	N.R.	N.R.	Pr/Po	WMGS (Illumina)	N.R.	Text + graphic	Similar with ground life	N.R.
3	Voorhies et al. (37)	RUS/ USA	Spaceflight	180–360	5	N.R.	N.R.	N.R.	Pr/Du/Po	16S rRNA gene (V4 hypervariable region) (Illumina)	OTUs	Text + graphic	N.R.	none
Subgroup 2: Isolation caused by spaceflight- or gravity simulators														
4	Brereton et al. (58)	RUS	Mars-500^1^-unit	520	6	29–40	0	25.5 (23.0–31.3)	Du	16S rRNA gene (V3-V4 hypervariable region) (Illumina)	ESVs	Text + graphic	Tinned foods similar to those used in ISS, by RUS/EUR/KOR/CHN firms, 15.1% protein, 33.4% fat, and 51.2% CH	*Streptococcus thermophilus* via yoghurt
5	Turroni et al. (59)					31.8 (27–38)			Pr/Du/Po		OTUs	Text		N.R.
6	Mardanov et al. (44)			510	5	28–38	N.R.	N.R.	Pr/Du/Po	16S rRNA gene (V3-V5 hypervariable region) (Pyrosequencing)	N.R.	Text + graphic		*Enterococcus faecium*, Eubikor, Vitaflor
7	Hao et al. (60)	CHN	Lunar Palace 1 (BLSS)	79–105	3	27–32	66. 6	21.3 ± 3 (19–24)	Pr/Du/Po	16S rRNA gene (V3-V4 hypervariable region) (Illumina)	OTUs	Text	Predesigned high-plant and high-fibre diet	N.R.
8	Li et al. (46)							N.R.				Graphic		
9	Meng et al. (61)	CHN	Lunar Palace 1 (BLSS)	63	4	26	50	N.R.	Du	WMGS (Illumina)	N.R.	Text	NASA astronauts’ dietary standards	N.R.
10	Chen et al. (62)	CHN	BLSS	60	4	23–27	50	18.5–22.9	Pr/Du/Po	WMGS (BGI-SEQ500 platform)	N.R.	Text	↑ CH, ↓ fat	N.R.
11	Dong et al. (63)	CHN	CELSS	180	4	26–36	25	18.6–24.6	Pr/Du	16S rRNA gene (V3-V4 hypervariable region) (Illumina)	OTUs	Text + graphic	in accordance with customs of Chinese population, 3 meals/d, main composition: CH, protein, fat, fiber	N.R.
Subgroup 3: Isolation in a natural, earth-bound habitat														
12	Jin et al. (64)	JPN	Antartica	60	6	37–55	0	N.R.	Pr/Du/Po	16S rDNA (T-RFLP, rtPCR)	OTUs	Text	N.R.	N.R.
13	Zhang et al. (65)	CHN	Sea voyage	30	42	25 ± 4.2	0	22.3 ± 2.7	Du	WMGS (Illumina)	N.R.	Graphic	Controlled, similar diet, Buffet style, min. 2 staple foods/d, 5 entrées/d, 2–3 fruit/d	*Lactobacillus casei Zhang*, *Lactobacillus plantarumP-8*, *Lactobacillus rhamnosus M9*, *Bifidobacterium lactis V9*, *Bifidobacterium lactis M8*
					40									no

“Isolation”: Isolated and confined extreme environments. “Length”: length of the isolation intervention, no pre/post surveys are considered. The sampling times are either before the start of the intervention (“Pre”), during the isolation itself (“During”), after the isolation (“Post”), or a combination of more than one of these times. “Additional intake”: intake of pro−/pre−/antibiotics during the isolation period. Control groups are not shown.

^1^Project to simulate a manned flight to Mars. 16S rDNA, 16S ribosomal Deoxyribonucleic acid; 16S rRNA, 16S ribosomal Ribonucleic acid; BLSS, Bioregenerative life support systems; CELSS, Controlled (or closed) ecological life-support systems; CH, carbohydrates; CHN, China; d, day; f, female; Du, During; JPN, Japan; N.R., not reported; Po, Post; Pr, Pre; rtPCR, reverse transcription polymerase chain reaction; RUS, Russia; T-RFLP, Terminal restriction fragment length polymorphism; USA, United States of America; WMGS, Whole metagenome shotgun sequencing. BMI, Body Mass Index; OTU, Operational taxonomic unit; ESV, exact sequence variants; KOR, Korea; EUR, Europe; NASA, National Aeronautics and Space Administration; ISS, International Space Station.

The 19 included studies ranged from 1964 to 2021 and data was mainly published by Asian researchers (*n* = 15), otherwise from American (*n* = 3) or by multiple institutions working together (*n* = 1). Shifts in microbial abundance as a consequence of residency in space (*n* = 4) or long-term confinement in natural, terrestrial habitats (*n* = 2) have been reported. However, the alterations of the human GI microbiota by isolation was mainly studied in spaceflight- and gravity simulation facilities (*n* = 13 via 9 different units). There are several publications on two of these experimental units: In the case of the Mars-500 experiment, 3 publications were identified (44, 52, 53) that differed partly regarding sample size, methodology and microbiota classification. Likewise, in case of the Chinese Lunar Palace 1 experiment, 3 publications (46, 54, 55) were found showing similar differences between each other. We thus examined a total of 142 participants being mainly men (exact number unclear). Most of the studies were conducted exclusively in men (9 studies), in 5 studies both sexes were included while in 5 studies sex was not reported. The median number of subjects involved in an intervention was 4, ranging from 1 to 82, covering the ages between 21 and 50 years (median age 30.4 years). Due to the natural or artificially created extreme conditions, all study subjects were of healthy condition and predominantly of normal weight (median BMI 22.6 kg/m^2^). The isolation intervention lasted on average 120 days (covering 30–520 days) and was accompanied by pre- and/or post-intervention measurements in >65% (*n* = 13). In 10 experiments, the diet was very tightly controlled and based on a typical space diet. Furthermore, 4 studies investigated the possibility of maintaining the GI microbiota composition by providing the volunteers with supportive probiotics or prebiotics for either regular or intermittent intake. Although all research groups collected stool samples to study the microbiota composition, no study reported stool frequency and/or consistency.

### 3.2. Summary of study outcomes

**Figure 2:**
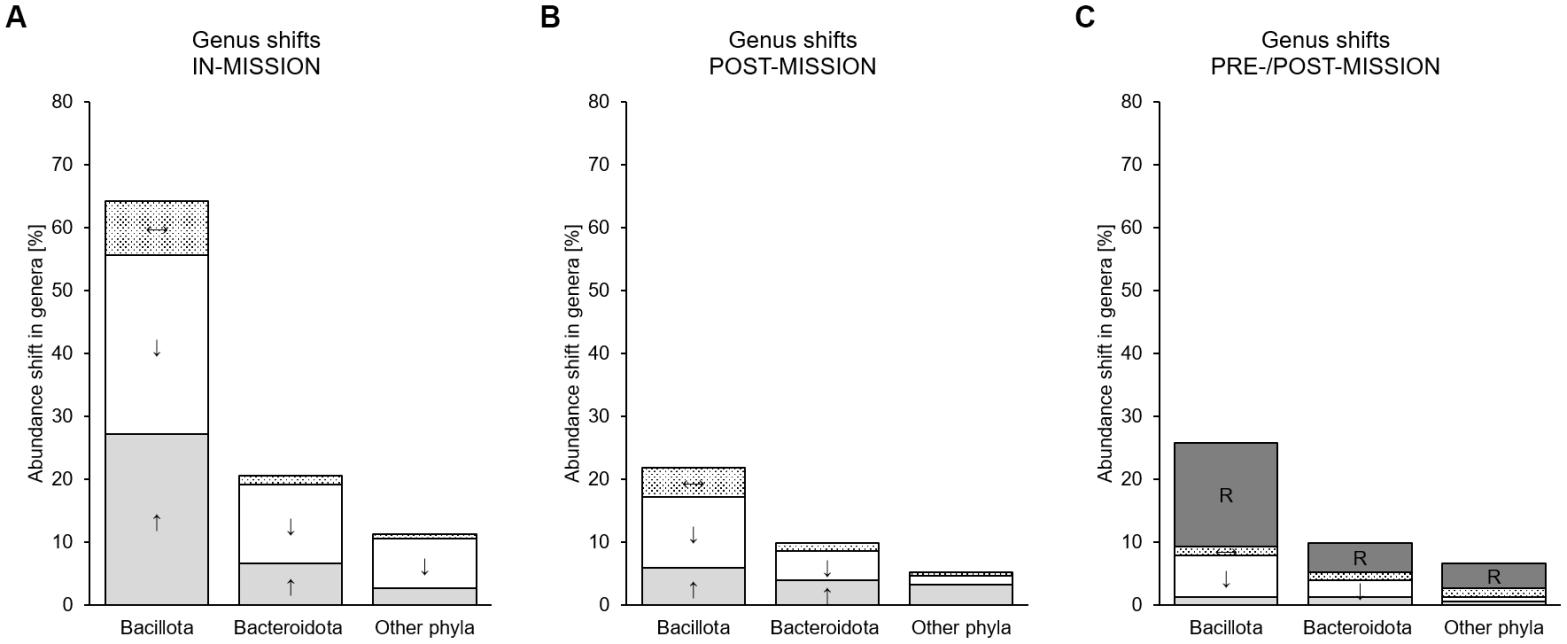
Microbial shifts in studies using molecular-based approaches for microbiota analysis [%] summarized in the genera-related phyla assuming that all shifts were reported, but everything else remained unchanged. Shifts that occured during isolation (in-mission, **(A)**, after isolation (post-mission, **(B)** and reported in the before-after comparison (pre/post-mission, **(C)** are presented. Summarized are Bacillota-, Bacteroidota- and other Phyla-related genera. ↓, white bar: Genus/species abundance [%] decreased during intervention; ↑, light grey bar: Genus/species abundance [%] increased during intervention; ↔, dotted bar: Genus/species abundance [%] remained unchanged; R, dark grey bar: Genus/species frequency [%] restored or partly restored to baseline levels.

A detailed overview of the in-mission outcomes for the single trials is presented in Table 3 for cultivation-based methods and Table 4 for molecular-based approaches. The outcomes across the studies are in the text and summarized in Figure 2. Further outcomes of pre- and post-mission are provided in the Supplements (Supplementary Table S1, S2).

**Table 3:** Study outcomes of microbiota analysis by cultivation method in-mission, outcomes are split according to corresponding isolation habitats.

Study ID	Author (year)	Subject	*Lactobacillus* spp.	*Bifidobacterium* spp.	*Escherichia* spp.	Other	
Subgroup 1: Isolation caused by space missions							
1	Lizko et al. (52)	Sojus 17/1	↓	N.R.	N.R.	N.R.	
		Sojus 17/2	↔	↔	↔	↔	
		Sojus 18/1	↑	↑	↑	Spore-forming bacteria *Proteus* spp.	↑ ↔
		Sojus 18/2	↑	↑	↑	Spore-forming bacteria *Proteus* spp.	↑ ↓
Subgroup 2: Isolation caused by spaceflight- or gravity simulators							
2	Chen et al. (53)	∑	↓	↓	N.R.	N.R.	
3	Cordaro et al. (54)	∑	↔	N.R.	N.R.	*Bacteroides* spp. *Enterococci* spp. Coliforms	↔ ↓ ↔
4	Gall and Riely (55)	1	↔	N.R.	↓ / ↑	*Klebsiella* spp. *Citrobacter* spp. *Shigella boydii* *Corynebacteria* spp.	↔ ↓ ↓ ↑
		2	↔	N.R.	↔	*Corynebacteria* spp.	↑
		3	↔	N.R.	↔	*Klebsiella* spp. *Corynebacteria* spp.	↑ ↑
		4	↔	N.R.	↑	*Klebsiella* spp. *Corynebacteria* spp.	↑ ↑
5	Rerberg et al. (56)	∑	↓	↓	N.R.	*Bacteroides* spp. *Clostridium perfringens*	↔ ↑
6	Shilov et al. (57)	∑	↓	↓	N.R.	*Clostridium perfringens*	↔
			↓	↓	N.R.	*Clostridium perfringens*	↑
			↔	↔	N.R.	*Clostridium perfringens*	↑

The arrows represent the direction of the microbial abundance shift. ↓: abundance reduction, ↑: abundance increase, ↔ no change in abundance detected. The study subjects can be reported individually, in groups or summarized for all study participants (∑). However, since this table summarizes multiple species within a genus (spp.), it is possible for the direction of shifts to vary among participants. N.R., not reported; spp.: multiple species of a genus.

**Table 4:** Bacterial diversity and microbial abundance shifts in-mission analyzed by molecular-based techniques, outcomes are summarized on phylum level.

Study			Microbial diversity shifts			Relative abundance shifts at phylum level						
Study ID	Author (Year)	Subject	α-D: Richness	α-D: Biodiversity	Community structure	Bacillota	Bacteroidota	Actinomycetota	Pseudomonadota	Verrucomicrobiota	Other	
Subgroup 1: Isolation caused by space missions												
1	Garret-Bakelman et al. (25)	∑	↔	↔	Dis.	*Taxa shifts have not been assigned by name.*						
2	Liu et al. (40)	A	↑		Dis.	↑	↓	↑	↑	↑	Fusobacteria Chlamydiae Tenericutes Aquificae	↔ ↓ ↓ ↔
		B	↓		Dis.	↓	↑	↓	↔	↑	Fusobacteria Chlamydiae Tenericutes Aquificae	↔ ↓ ↔ ↓
3	Voorhies et al. (37)	∑	↑	↑	Dis.	↔	↔	↔	↔	↔	↔	
Subgroup 2: Isolation caused by spaceflight- or gravity simulators												
4	Brereton et al. (58)	∑	↔	↔	N.R.	↔	↔	↔	↔	↔	↔	
5	Turroni et al. (59)	∑	N.R.	N.R.	Sim.	↔	↔	↔	↔	↔	↔	
6	Mardanov et al. (44)	A	N.R.	N.R.	N.R.	↔	↔	↔	↔	↔	↔	
		B	N.R.	N.R.	N.R.	↔	↔	↔	↔	↔	Fusobacteria	↓
		C	N.R.	N.R.	N.R.	↔	↔	↔	↔	↓	↔	
		D	N.R.	N.R.	N.R.	↔	↔	↑	↔	↔	↔	
		E	N.R.	N.R.	N.R.	↔	↔	↔	↔	↔	↔	
7	Hao et al. (60)	∑	↑		Dis. *	↑	↓	↔	↔	↔	↔	
8	Li et al. (46)	∑	N.R.	N.R.	N.R.	↔	↔	↔	↔	↔	↔	
9	Meng et al. (61)	∑	N.R.	N.R.	N.R.	↔	↔	↔	↔	↔	↔	
10	Chen et al. (62)	∑	N.R.		Dis. *	↔	↔	↔	↔	↔	↔	
		f	↓			↔	↔	↔	↔	↔	↔	
		m	↑			↔	↔	↔	↔	↔	↔	
11	Dong et al. (63)	∑	↓	↓	N.R.	↓	↑	↔	↔	↔	Fusobacteria	↑
Subgroup 3: Isolation in a natural, earth-bound habitat												
12	Jin et al. (64)	∑	N.R.	N.R.	N.R.	↔	↔	↔	↔	↔	↔	
13	Zhang et al. (65)	Plac.	↔	↔	Dis.	↔	↔	↔	↔	↔	↔	
		Pro.	↔	↔	Sim.	↔	↔	↔	↔	↔	↔	

The arrows represent the direction of the microbial abundance shift. ↓: abundance reduction, ↑: abundance increase, ↔ no change in abundance detected, *: Trend to converge. The study subjects can be reported individually, in groups or summarized for all study participants (∑). α-D, alpha-Diversity; Dis, dissimilar; f, female; m, male; N.R., not reported; Plac., Placebo group; Pro., Group taking additional probiotics during the intervention; Sim., similar.

In the cultivation studies, changes in *Lactobacillus*, *Bifidobacterium*, (potentially pathogenic) *Escherichia*, spore-forming bacteria, *Proteus, Bacteroides*, *Enterococcus*, *Klebsiella, Citrobacter*, *Shigella, Corynebacterium* and, in simulated emergency situations, changes in *Clostridium perfringens* were detected. One study found that the Bacteroides were the most numerous and stable, whereas severe fluctuations occurred in the transit microbiota (mentioned *Staphylococcus* and yeast) (56). Another study also reported alterations, which, however, tended to return normal after the end of the confinement (58). Finally, one study examined the aerobic and anaerobic flora in four young men over a six-week period. As the subjects had been on the space-diet for a longer period of time, differences in both the aerobic and anaerobic flora began to appear. Seven new types of organisms became prevalent, which had not been described before, mostly gas-forming, black slime-producing, and proteolytic organisms. It was assumed that the specialized space-diet (not further reported) was a contributing factor (59). Due to the different cultivation conditions, the cultivated bacterial groups observed were heterogeneous between studies.

The introduction of molecular-based approaches led to a better assessment of the microbiota allowing for a comprehensive description of microbial communities. Out of 13 studies, alpha diversity was reported to be reduced (richness: *n* = 3; biodiversity *n* = 3), to remain stable (richness: *n* = 4; biodiversity *n* = 4) or to increase (richness: *n* = 4; biodiversity *n* = 4). Richness and biodiversity were not reported separately in 3 studies (40, 56, 60). More dissimilarities were reported in microbial community structure (*n* = 7) with a tendency towards convergence observed repeatedly (54, 60). However, in a study that reported the differences between control subjects, the scale of microbiota changes in microbial diversity in the test subject during isolation was relatively small (25).

At the phylum level abundances predominantly remained stable following the confinement; in all but 4 of the 13 included studies (*n* = 14 participants) shifts in abundances were observed.

Changes reported at genus (Table 5) and species level (Supplementary Table S3) were not consistent across studies. Even more, *Bacteroides* (spp.), *Eubacterium* (spp.), *Faecalibacterium* (spp.), *Lactobacillus* (spp.), *Prevotella* (spp.), *Alistipes* (spp.), Blautia (spp.), *Lachnospira*, *Ruminococcus* (spp.), *Bifidobacterium* (spp.) and *Clostridium* (spp.) were reported to be either increasing or decreasing in their relative abundances in-mission. Across all studies, abundance shifts were predominantly associated with the phyla Bacillota (67.5%) and Bacteroidota (20.5%, Figure 2). Regardless of the taxonomic ranking, two studies reported no shifts at all (25, 55). The proportions of increasing and decreasing phyla decrease in Figures 2B,C due to the diminishing number of reporting studies.

**Table 5:** Study outcomes for each subgroup at genus level for all studies and all sampling times (pre-intervention, during-intervention, post-intervention) for molecular-based analyses.

Phylum	Genus	Space									Simulation									Natural, earth-bound								
		In-mission			Post-mission			Pre/post			In-mission			Post-mission			Pre/post			In-mission			Post-mission			Pre/post		
Verrucomicrobiota	*Akkermansia* (spp.)		↓^3^						R^3^						↓^5^													
Bacteroidota	*Alistipes* (spp.)	**↑**^2^				**↓**^2^		↑^2^		↔^2^	↑^6^	↓^11^				↔^6^		pR^6^			↓^13^							
Bacillota	*Anaerostipes* (spp.)											↓^4,10^																
Bacteroidota	*Bacteroides* (spp.)	**↑**^2^	**↓**^2^		**↑**^2^	**↓**^2^		↑^2^	↓^2^		↑^4,5^	↓^6,10,11^	↔^6^	↑^6^	↓^6^	↔^6^		pR^6^			↓^12,13^		↑^12^				R^12^	
Actinomycetota	*Bifidobacterium* (spp.)		**↓**^2^	**↔**^2^	**↑**^2^		**↔**^2^					↓^10^								↑^12,13^	↓^12^		↑^12^				R^12^	
Bacillota	*Blautia* (spp.)	**↑**^2^		**↔**^2^	**↑**^2^	**↓**^2^		↑^2^		↔^2^	↑^7^	↓^4^			↓^7^													
Bacteroidota	*Butyricimonas*											↓^11^																
Bacillota	*Christensenellaceae*										↑^4^																	
Bacillota	*Clostridium* (spp.)	**↑**^2^		**↔**^2^		**↓**^2^			↓^2^			↓^10^								↑^12^	↓^12,13^		↑^12^	↓^12^				
Bacillota	*Coprococcus* (spp.)								↓^3^				↔^5,6^					pR^6^										
Bacillota	*Dialister*											↓^11^	↔^6^		↓^6^	↔^6^		pR^6^										
Bacillota	*Dorea* (spp.)		**↓**^3^						R^3^				↔^5^															
Pseudomonadota	*Enterobacter* (spp.)											↓^10^																
Bacillota	*Enterobacteriales*																				↓^12^			↓^12^				
Pseudomonadota	*Escherichia* (spp.)		**↓**^2^		**↑**^2^	**↓**^2^			↓^2^	↔^2^		↓^10^																
Bacillota	*Eubacterium* (spp.)	**↑**^2^	**↓**^2,3^			**↓**^2^		↑^2^	↔^2^	R^3^																		
Bacillota	*Faecalibacterium* (spp.)	**↑**^3^	**↓**^2^	**↔**^2^		**↓**^2^			↓^2^	R^3^	↑^7,11^	↓^4,5,10^			↓^7^			pR^6^										
Bacillota	*Flavonifractor*																				↓^13^							
Bacillota	*Fusicatenibacter*	**↑**^3^							R^3^																			
Bacillota	*Kineothrix*										↑^4^																	
Pseudomonadota	*Klebsiella*																				↓^13^							
Bacillota	*Lachnospira*	**↑**^3^							R^3^		↑^7^	↓^4,11^			↓^7^													
Bacillota	*Lachnospiraceae*	**↑**^3^							R^3^		↑_4,10_																	
Bacillota	*Lactobacillus* (spp.)	**↑**^2^	**↓**^2^			**↓**^2^	**↔**^2^		↓^2^			↓^4^								↑^13^								
Bacillota	*Lactococcus*																				↓^13^							
Fusobacteria	*Leptotrichia*		**↓**^3^						R^3^																			
Bacillota	*Megamonas*										↑^6^			↑^6^				pR^6^										
Bacillota	*Megasphaera*		**↓**^3^						R^3^																			
Bacteroidota	*Parabacteroides*											↓^11^																
Pseudomonadota	*Parasutterella*	**↑**^3^							R^3^																			
Bacillota	*Phascolarctobacterium*											↓^6^	↔^6^	↑^6^	↓^6^	↔^6^		pR^6^										
Bacteroidota	*Prevotella* (spp.)	**↑**^2^	**↓**^3^			**↓**^2^		R^3^	↓^2^	↔^2^	↑^10,11^	↓^6,7^	↔^6^	↑^6,7^	↓^6^		↑^6^	↓^6^	R		↓^13^							
Bacillota	*Pseudobutyrivibrio*		**↓**^3^						R^3^		↑^11^																	
Bacillota	*Roseburia* (spp.)	**↑**^2^				**↓**^2^			↓^2^			↓^4^									↓^13^							
Bacillota	*Ruminiclostridium*	**↑**^3^							R^3^																			
Bacillota	*Ruminococcaceae*										↑^4^																	
Bacillota	*Ruminococcus* (spp.)		**↓**^3^						↓^3^		↑^4^	↓^4,11^																
Bacillota	*Streptococcus*		**↓**^3^						R^3^		↑^4^									↑^13^	↓^13^							
Bacillota	*Subdoligranulum*										↑^11^																	
Bacillota	*Veillonella*		**↓**^3^						R^3^		↓^10^																	

Microbiota abundance fluctuations/continuities identified at genus level in (1) Subgroup 1: Isolation in Space [Study ID 1–3, molecular-based approaches (Tables 2, 4)], (2) Subgroup 2: Isolation in a simulation unit [Study ID 4–11, molecular-based approaches (Tables 2, 4)], and (3) Subgroup 3: Natural earth-bound isolation [Study ID 12–13, molecular-based approaches, (Tables 2, 4)] either (i) through the intervention compared to baseline (in-mission) or (ii) after the isolation period compared to the intervention (post-mission) or (iii) after the isolation compared to baseline (pre−/post-mission).

↓, Relative abundance decreased; ↑, Relative abundance increased; ↔, No significant shifts in relative abundance. R, Restored back to baseline level. pR, partially restored back to baseline. Studies have reported only on genus level or also including some species (spp.).

No isolation type provided an indication that the intensity of a change is related to the length of the confinement.

### 3.3. Subgroup analyses

Due to the heterogeneity in outcome comparisons and microbiota analysis techniques used, it was not feasible to summarize the findings clearly in figures. This led to the breakdown into groups and subgroups, as mentioned in the methods section.

#### 3.3.1. Subgroup analysis 1: GI microbiota under isolation conditions in space

One study cultivated and analyzed the samples collected during a space mission lasting 30 and 63 days each with 2 astronauts independent of one another (61). Most of the bacteria analyzed remained stable in the pre/post comparison except for a decline in *Lactobacillus* and a reduction of *Escherichia* and *Proteus* in two individuals.

Three other studies (25, 37, 40) published data from space samples (*n* = 8 astronauts) using molecular-based techniques to analyze microbiota changes.

Beta-diversity has consistently been described as dissimilar in-mission (25, 37, 40), while both richness and evenness demonstrated a rather heterogeneous distribution. In one study, Shannon’s alpha diversity and richness significantly increased in space and returned to their baseline preflight levels after crew members returned to earth (37). Another study reported that alpha diversity at genus level did not fluctuate significantly, but the fluctuations between each subject were dissimilar (40).

Phyla abundance remained rather stable; only Liu et al. (40) observed changes during and after the isolation as well as significant changes in the pre−/post-comparison. Over a 35-day space mission, two study subjects were isolated and examined. The phyla of the two study subjects conflicted; when a phylum of one subject changed, its equivalent did not change or in the other direction. Bacillota and Bacteroidota displayed a remarkable antagonistic behavior in both subjects (Table 4). Additionally, two studies (37, 40) reported data on shifts at the genus level for *n* = 7 subjects (Table 5; Supplementary Table S3). Most changes in the abundance of genera were related to Bacillota. Concurrent divergent shifts to either higher or lower abundances of the same genera were described, confirming heterogeneity. Almost all shifts were abolished after the spaceflight, indicative of resilience. Further results are very heterogeneous and are therefore not discussed here.

#### 3.3.2. Subgroup analysis 2: GI microbiota under isolation conditions in experimental facilities

Isolations conducted in a controlled artificial environment or similar unnatural unit using cultivation techniques predominantly identified *Lactobacillus*. There was either no change or a drop in *Lactobacillus*. *Bacteroides* was characterized to be one of the most stable microbial groups. *Bifidobacterium* spp. was cultivated less commonly, however, most subjects showed a reduction of the genus across studies. Inconsistent changes were observed in *Escherichia coli* and *Klebsiella* and unique shifts in Coliforms and *Enterococcus*, *Corynebacterium*, *Shigella boydii* and *Citrobacter*. In the course of the experiment, some subjects showed a prominent increase in toxigenic strains of *Clostridium perfringens*, which is suspected of being related to lipid metabolism.

Eight other studies published data from simulation unit experiments using molecular-based approaches to analyze microbiota changes (Table 4). For this subgroup, it is hardly possible to comment on diversity, as it was either rarely reported or, if reported, did not give a conclusive trend. Several shifts occurred during isolation (Supplementary Table S3) in *Bacteroides* (predominantly reducing), *Faecalibacterium* (rather reducing) and *Prevotella* (no directional tendency). *Lachnospira* and *Ruminococcus* also frequently showed significant changes in their abundance during the intervention, but these were very heterogeneous with tendencies towards reduction. One study (44) further reported a follow-up time and compared the pre- and post-measurements. Almost all of the investigated genera showed a return to the baseline proportions or they were at least partly restored to baseline levels.

#### 3.3.3. Subgroup analysis 3: GI microbiota under isolation conditions in natural isolated habitats

There was no data from natural habitats using cultivation methods.

Using molecular-based approaches, two studies provided data from natural isolated habitats. During a period of 2 months, study subjects (*n* = 6) were stationed on a research station in Antarctica for 3 months in the study of Jin et al. (62). The study results were presented in terms of Operational Taxonomic Units (OTUs), showing mainly changes in the abundance of *Bacteroides* and *Bifidobacterium* species, decreasing during the expedition in four subjects and increasing in two subjects. Furthermore, a comparison between pre- and post-residence in Antarctica was possible for OTUs, with predominantly *Bacteroides* spp. showing no significant changes while all *Bifidobacterium* spp. increased, decreased, or remained stable. The study found that the participants had interindividual variability in their fecal microbiota, and cold, stress and changes in food intake were possible factors affecting their microbiota.

The study by Zhang et al. (63) determined the effects of probiotics on sailors (*n* = 82) over the course of a 30-day cruise. Here, it needs to be considered, that this study is different from the other with extreme environments due to the large samples size. Probiotics were administered to some but not all of the sailors in this study. None of the described study models caused an increase or decrease in the abundance of phyla; even with the administration of probiotics to sailors, no differences were found. However, the administration of probiotics indicated that the impact of a long sea voyage on the intestinal microbiota were significant as beta diversity distances were significantly larger in the placebo group than in the probiotic group. The result was confirmed by alpha-diversity, which showed no significant differences among groups, but a sharp decline between probiotic and placebo groups while on mission. Only shifts in abundances at the species level were found, whereby the ratio between declines and increases was rather balanced with tendencies towards more increases.

### 3.4. Similarities and differences across habitats

**Figure 3:**
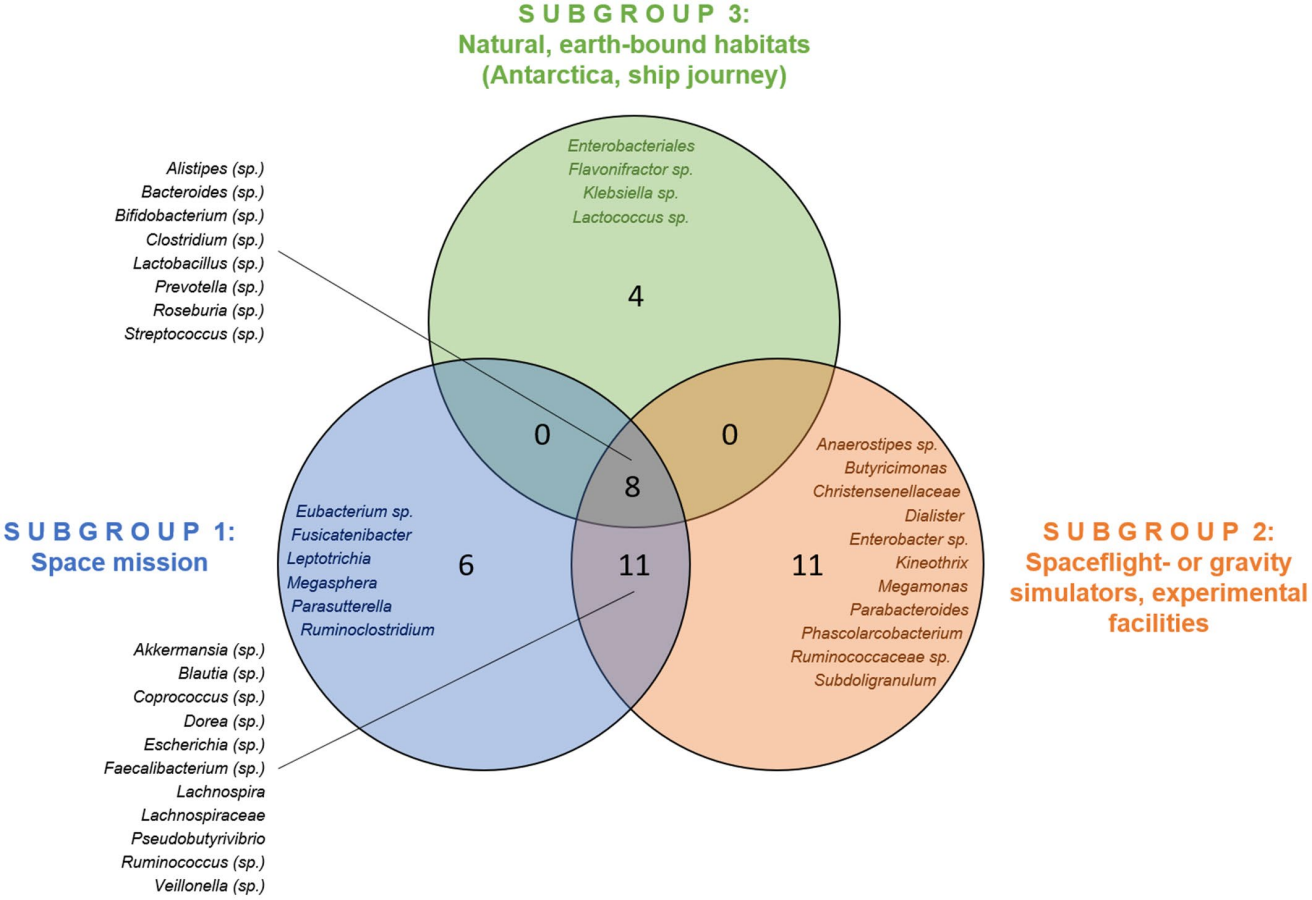
Overlap of reported changes in genera across the habitats studied. Species-specific changes (sp.) are also mentioned.

Studies in space and simulation units were more similar in both genera and species profiles, while in the natural habitats the shifts were reported to be more diverse (Table 5; Figure 3). However, 8 shifts in genera or genus-specific species were shared by all three habitats. Space and simulation experiments shared shifts in 11 genera or genus-specific species.

There was a high degree of heterogeneity throughout all habitats during the intervention. It has been observed that neither species that exhibited abundance shifts across all three habitats showed the same trend. Only some *Streptococcus* species shifted rather consistently in abundance during the intervention (Supplementary Table S3).

### 3.5. Further outcomes

Besides GI microbiota responses to extreme environmental conditions, significant reductions in body mass (25, 54, 55) as well as energy intake (64) were found. In line with these results are blood values for metabolism. Li et al. (46) and Hao et al. (54) reported that the participants’ health parameters (heart rate, blood pressure and BMI) were within the normal range throughout the study.

Furthermore, significant changes in several physiological parameters were observed, accompanied by an increase in inflammatory parameters (37, 53), changes in urinary metabolites (65), a decrease in mineral bone density and in muscle turnover (65), changes in respiratory minute volume (56), telomere elongation (25) as well as disrupted glucose metabolism (53), affected antibiotic resistance genes (40), genome instability, DNA methylation in immune and oxidative stress-related pathways (25) and finally a reduction in 25-hydroxyvitamin D levels (64). However, none of the participants demonstrated symptoms of diseases associated with considerable changes in the composition of the microbiota (44). After the isolation, 25-hydroxyvitamin D levels (64), but also mean telomere length and global gene expression converged to pre-flight levels, with an increased number of short telomeres observed and the expression of some genes still disturbed (25). However, it remains unclear if these changes are in relation to confinement or in association to microbiota shifts.

In contrast, some psychological parameters were reported to be affected, e.g., an increase in the abundance of *Faecalibacterium* spp. correlating negatively with mood (46). Scores of stress and anxiety seemed to be lower (63), except when in the situation of absence of natural light, where the incidence of anxiety and depression increased (55). After the isolation period of 340 days, a cognitive decline was observed (25).

However, many studies reporting different aspects of human biology and health outcomes due to isolation and confinement have been published elsewhere (42, 66) which is why only the changes that have been reported in the context of our investigation are mentioned here.

### 3.6. Risk of bias

The risk of bias for the studies included in the review conducted via the ROBINS-I checklist is presented in Table 6 for all studies. Among the 19 non-RCTs, 1 was considered to have critical risk of bias due to confounding bias and 4 were at serious risk of bias. It seems that the older studies have this increased risk. Only one study was included providing a low risk of bias. A moderate risk of bias was commonly attributed to bias in selection of the reported results, which is why the highest number of studies provides a moderate risk (68%).

**Table 6:** ROBINS-I risk of bias for all studies.

Study	Bias due to confounding	Bias in selection of participants into the study	Bias in classification of interventions	Bias due to deviations from intended interventions	Bias due to missing data	Bias in measurement of outcomes	Bias in selection of the reported result	Overall bias
Brereton et al. (53)	Low	Low	Low	Low	Low	Low	Moderate	**Moderate**
Chen et al. (65)	Low	Low	Low	Low	Low	Low	Moderate	**Moderate**
Chen et al. (60)	Low	Low	Low	Low	Low	Low	Moderate	**Moderate**
Cordaro et al. (58)	NI	Low	Low	Low	Moderate	Serious	Moderate	**Serious**
Dong et al. (64)	Low	Low	Low	Low	Low	Low	Moderate	**Moderate**
Gall and Riely (59)	Critical	Low	Low	Low	Low	Moderate	Moderate	**Critical**
Garret-Bakelman et al. (25)	Moderate	Low	Low	Moderate	Low	Low	Moderate	**Moderate**
Hao et al. (54)	Low	Moderate	Low	Low	Low	Low	Moderate	**Moderate**
Jin et al. (62)	Low	Low	Low	Low	Low	Low	Moderate	**Moderate**
Li et al. (46)	Low	Serious	Low	Low	Low	Low	Moderate	**Serious**
Liu et al. (40)	Low	Low	Low	Low	Low	Low	Low	**Low**
Lizko et al. (61)	Low	Low	Low	Low	Serious	Moderate	Moderate	**Serious**
Mardanov et al. (44)	Serious	Low	Serious	Moderate	Low	Moderate	Moderate	**Serious**
Meng et al. (55)	Low	Low	Low	Low	Low	Low	Moderate	**Moderate**
Turroni et al. (52)	Low	Low	Low	Low	Low	Low	Moderate	**Moderate**
shilov et al. (57)	Low	Low	Low	Low	Low	Low	Moderate	**Moderate**
Rerberg et al. (56)	NI	NI	Low	Low	Low	Low	Moderate	**Moderate**
Voorhies et al. (37)	Low	Low	Low	Low	Low	Low	Moderate	**Moderate**
Zhang et al. (63)	Low	Low	Low	Low	Low	Low	Moderate	**Moderate**

A risk of bias is classified as “Low risk” (green coloured), “Moderate risk” (yellow coloured), “Serious risk” (orange coloured), or “Critical risk” of bias (red coloured). Some risks of bias could not be assessed due to lack of information and are therefore marked with “No information (NI)” (grey coloured).

## 4. Discussion

This review included 19 articles and examined to what extent the GI microbiota changes during and after a stay in an isolated, antigen-limited or poor environment. Overall, changes in GI microbiota were observed and reported following confinement; however, the type and extent of the reported changes varied between studies. The data do not point to a clear direction, but nevertheless, in line with the assumptions, indicate to some extent trends in changes.

This review affirms that experimental isolation results in a decrease in alpha diversity (richness and biodiversity) and does not lead to increased similarity in the GI microbiota among subjects. A systematic review examining the human GI microbiota during long-term space missions confirms our results suggesting that space travel may lead to microbial dysbiosis and metabolic changes in the human gut, including a drop in alpha diversity (67). Similarly, a recent experimental study investigating the impact of an enhanced spaceflight diet on 16 subjects over a 45-day closed chamber mission found that subjects eating a standard diet demonstrated a decline in Shannon’s alpha biodiversity and richness during the mission, but recovered fully by the end of it (68). However, these effects are not evident and consistent across all participants of our review, as an increase in richness and biodiversity has been observed in some test persons, which is suspected in relation to an increased dietary fibre intake (54) or merely due to individual differences (40). The increase of alpha richness and biodiversity is discussed extensively with regard to health protection (37, 54) since a biodiverse ecosystem is more resistant and resilient to perturbations and has a lot more functional redundancy (69). In the studies we know of involving additional administration of probiotics (53, 63), both richness and biodiversity were rather preserved. However, in some studies (40, 54) alpha-diversity is not reported separately for richness and biodiversity, which can limit our understanding of the overall diversity of the studied ecosystem. Alpha-diversity is a measure of diversity within a particular habitat or community. By reporting alpha-diversity as a single value without distinguishing between richness and evenness, researchers may miss important patterns in the distribution of diversity within their study system.

In contrast, hardly any significant changes in community structure were observed. Overall, the community structures remained heterogeneous and dissimilar between individuals, although a convergence of the GI microbiota was reported in several cases. Surprisingly, the effect of convergence was stronger in placebo groups compared to groups supplemented with probiotics (63). This finding is in line with more recent outcomes regarding maintaining GI microbiota eubiosis by probiotics (70). Furthermore, it is speculated that an enhanced spaceflight diet containing increased quantity and variety of fruits, vegetables, fish and other foods rich in flavonoids and omega-3 fatty acids, preserves community structure in the comparative analysis between pre- and in-mission time points (68). To potentially control or better mitigate negative effects better in the future, a diet-based therapy including higher contents of fibre could possibly provide an effective treatment (71, 72). We only know from Hao et al. (54), that a high-fibre diet has been used for the experimental study. The participants’ community structure remained dissimilar, however, a convergence was apparent, sup-porting the possible importance of fibre for GI microbiome homeostasis. Previous studies have even highlighted the important effect of diet through the results of their experiments (59). Besides, the administration of probiotic supplements could be considered as another approach to maintain the gut microbiome homeostasis during the stay in isolated environments (70).

Changes in the abundance of a few specific microbiota taxa were reported, supporting our first research question; however, changes reported at the genus and species level were not consistent across studies and at the phylum level abundances remained stable during the mission. Most changes occurred in Bacillota and Bacteroidota, which is consistent with other studies describing microbial changes (14, 15, 17). Both other external and internal parameters influencing the microbiota may induce changes at this phylum level. The reason for this is the high relative abundance of these phyla in the individual core microbiome (8, 9). In more detail, notable changes were reported multiple times for genera *Alistipes*, *Bacteroides*, *Bifidobacterium*, *Faecalibacterium* and *Prevotella*, although there was no clear direction of development for these either. Alterations in intestinal genus levels is often present in case of intestinal microbiota dysbiosis, even more, a dysbiotic abundance of those genera is associated with several diseases (73, 74). Furthermore, the human microorganism ecosystem in an antigen-poor environment, such as a spacecraft, space- or microgravity simulators or Antarctica, has the potential for a loss of the barrier function protecting against pathogens, which, together with a potentially weakened immune system during spaceflight, poses the risk of more severe infection during long-term spaceflight (42). However, none of our included participants demonstrated symptoms of diseases associated with considerable changes in the composition of the microbiota. Thus, it can be assumed that restructuring of the taxonomic composition occurred in their intestinal ecosystems, reflecting their individual responses to the conditions of the experiment and a new balanced community was formed (44). However, the sample sizes are overall quite low so it is speculative but it can be suspected that the baseline microbiota could play a role, some being more resistant than others to change upon isolation.

A detailed analysis and thus an attempt to compare the results was predominantly possible in the studies using molecular-based approaches. Due to the difficulty in cultivating many types of gut bacteria in laboratory conditions, studies of the GI microbiome have been restricted in the past (75). We have gained a greater understanding of the composition, diversity and roles of the gut microbiome in human health and disease with the development of molecular-based metagenomics (76). A summary of the changes that were carried out via the cultivation method seems even more difficult due to this. However, there appears to be a tendency towards just as unspecific shifts as in the studies that were analyzed using molecular-based approaches. A systematic literature search reported that although still not conclusive, there is a wealth of evidence suggesting that space travel may lead to microbial dysbiosis and metabolic changes in the human gut, including a drop in alpha diversity and changes in gene expression of culturable bacteria (67).

Our second research question aimed to investigate the diversity and abundance of the GI microbiota after experimental exposure. Most of the included studies report a partial recovery after the mission regarding both diversity analysis and microbial abundance within a few weeks, although, studies investigating the recovery were limited. Overall, the data show, that constant environmental factors can partly influence the individual GI microbiota. However, the great interindividual variety remains throughout the experiments which can be attributed to intrinsic factors such as age, genetics and immune system, constantly shaping the GI microbiota persistently. Although resilience of the microbiota following stress such as a course of antibiotics has been commonly observed (77, 78), it is now suspected that harsh or chronic stress such as inflammation could lead to an altered host-microbes relationship associated with a loss of resilience (79). Our analysis would indicate that stress conditions imposed by spaceflight or its simulations does not push the host-microbes system beyond its ecological robustness but rather allows expression of resilience.

### 4.1. Strengths and weaknesses of the systematic review

Overall, this systematic review has several strengths and limitations. A clear strength is the methodological approach taken according to PRISMA and Cochrane criteria. In order to obtain as broad as possible a knowledge of the current data situation, a very specific search term was used which was superior to broader search terms; however, only 19 articles could be included in the analysis. Despite clear eligibility criteria, the heterogeneity of the studies was high at the methodology (starting from sample preparation and processing) and descriptive levels. To counter this problem, subgroup analyses were performed which reduced heterogeneity to some degree. Despite differences in analysis techniques, habitats, study designs and frameworks for well-conducted studies, all studies were rather highly controlled, which is also reflected in the risk of bias. Here, the ROBINS-I tool for assessing risk of bias in non-randomized studies of interventions, recommended by the Cochrane Handbook, was used.

One of the main issues in the studies reviewed was the extremely low sample sizes, making it challenging to conduct quantitative analyses at the individual study level. Additionally, there were inconsistencies in the study protocols and characteristics, making comparisons between studies difficult. To address these limitations, future studies should aim to increase their sample sizes to enhance statistical power. Furthermore, most of the studies did neither analyze immunological/biochemical parameters in parallel to the microbiota analysis or, the data were published separately and not reported in context. To better understand the effects of long-term isolation on the human GI microbiota, researchers should consider internal and external factors, such as nutrition, genetics, and the immune system. This will enable a clearer differentiation between the effects of isolation and those stemming from other variables. Finally, this systematic review is the first of its kind, providing new insights into the effects of isolation on the human GI microbiota.

## 5. Conclusion

Overall, our review highlights the complexity of the relationship between the human GI microbiota and its environment. While extreme conditions can affect the composition of the GI microbiota, the internal factors that have shaped the microbiota over time appear to be the primary drivers of its composition and function in response to isolation in antigen-deprived conditions. Maintaining and/or strengthening the host’s fitness and immunity through diet, pre- and probiotics, and favorable lifestyle factors may have a positive impact on the human GI microbiota, especially under extreme conditions, and promote GI health and prevent disease.

## Data availability statement

The original contributions presented in the study are included in the article/Supplementary material, further inquiries can be directed to the corresponding author.

## Author contributions

IM and BK: conceptualization. BK and CS: methodology. BK, CS, and JK: formal analysis. BK (80%) and IM (20%): writing–original draft preparation. IM, PE, JP, JD, and CL: writing–review and editing. BK: visualization. IM: supervision. All authors have read and agreed to the published version of the manuscript.

## Funding

IM acknowledges support by the German Aerospace Center (DLR), grant number 50WB1920. This publication work was supported in part by the European Commission in the context of ERC-2017-AdG N°788191- *Homo symbiosus*. All authors thank the Open Access Publishing Fund of Tübingen University for support.

## Conflict of interest

The authors declare that the research was conducted in the absence of any commercial or financial relationships that could be construed as a potential conflict of interest.

## Publisher’s note

All claims expressed in this article are solely those of the authors and do not necessarily represent those of their affiliated organizations, or those of the publisher, the editors and the reviewers. Any product that may be evaluated in this article, or claim that may be made by its manufacturer, is not guaranteed or endorsed by the publisher.

## References

[1] Belkaid Y Hand TW. Role of the microbiota in immunity and inflammation. Cells. (2014) 157:121–41. doi: 10.1016/j.cell.2014.03.011 PMC4056765 24679531

[2] Hooper LV Littman DR Macpherson AJ. Interactions between the microbiota and the immune system. Science. (2012) 336:1268–73. doi: 10.1126/science.1223490, PMID: 22674334 PMC4420145

[3] Nicholson JK Holmes E Kinross J Burcelin R Gibson G Jia W. Host-gut microbiota metabolic interactions. Science. (2012) 336:1262–7. doi: 10.1126/science.1223813 22674330

[4] Cryan JF O’Mahony SM. The microbiome-gut-brain axis: from bowel to behavior: from bowel to behavior. Neurogastroenterol Motil. (2011) 23:187–92. doi: 10.1111/j.1365-2982.2010.01664.x 21303428

[5] Wang B Yao M Lv L Ling Z Li L. The human microbiota in health and disease. Engineering. (2017) 3:71–82. doi: 10.1016/J.ENG.2017.01.008

[6] Viaud S Saccheri F Mignot G Yamazaki T Daillère R Hannani D. The intestinal microbiota modulates the anticancer immune effects of cyclophosphamide. Science. (2013) 342:971–6. doi: 10.1126/science.1240537, PMID: 24264990 PMC4048947

[7] Weiss GA Hennet T. Mechanisms and consequences of intestinal dysbiosis. Cell Mol Life Sci. (2017) 74:2959–77. doi: 10.1007/s00018-017-2509-x 28352996 PMC11107543

[8] Tap J Mondot S Levenez F Pelletier E Caron C Furet J-P. Towards the human intestinal microbiota phylogenetic core. Environ Microbiol. (2009) 11:2574–84. doi: 10.1111/j.1462-2920.2009.01982.x, PMID: 19601958

[9] Qin J Li R Raes J Arumugam M Burgdorf KS Manichanh C. A human gut microbial gene catalogue established by metagenomic sequencing. Nature. (2010) 464:59–65. doi: 10.1038/nature08821, PMID: 20203603 PMC3779803

[10] Jost L. Partitioning diversity into independent ALPHA and BETA components. Ecology. (2007) 88:2427–39. doi: 10.1890/06-1736.1, PMID: 18027744

[11] Tuomisto H. A diversity of beta diversities: straightening up a concept gone awry. Part 1. Defining beta diversity as a function of alpha and gamma diversity. Ecography. (2010) 33:2–22. doi: 10.1111/j.1600-0587.2009.05880.x

[12] Turnbaugh PJ Hamady M Yatsunenko T Cantarel BL Duncan A Ley RE. A core gut microbiome in obese and lean twins. Nature. (2009) 457:480–4. doi: 10.1038/nature07540, PMID: 19043404 PMC2677729

[13] Koenig JE Spor A Scalfone N Fricker AD Stombaugh J Knight R. Succession of microbial consortia in the developing infant gut microbiome. Proc Natl Acad Sci U S A. (2011) 108:4578–85. doi: 10.1073/pnas.1000081107, PMID: 20668239 PMC3063592

[14] Seganfredo FB Blume CA Moehlecke M Giongo A Casagrande DS Spolidoro JVN. Weight-loss interventions and gut microbiota changes in overweight and obese patients: a systematic review: weight-loss impact on gut microbiota. Obes Rev. (2017) 18:832–51. doi: 10.1111/obr.12541, PMID: 28524627

[15] Mack I Cuntz U Grämer C Niedermaier S Pohl C Schwiertz A. Weight gain in anorexia nervosa does not ameliorate the faecal microbiota, branched chain fatty acid profiles and gastrointestinal complaints. Sci Rep. (2016) 6:26752. doi: 10.1038/srep26752, PMID: 27229737 PMC4882621

[16] Cook J Lehne C Weiland A Archid R Ritze Y Bauer K. Gut microbiota, probiotics and psychological states and behaviors after bariatric surgery—a systematic review of their interrelation. Nutrients. (2020) 12:2396. doi: 10.3390/nu12082396, PMID: 32785153 PMC7468806

[17] Palmisano S Campisciano G Silvestri M Guerra M Giuricin M Casagranda B. Changes in gut microbiota composition after bariatric surgery: a new Balance to decode. J Gastrointest Surg. (2020) 24:1736–46. doi: 10.1007/s11605-019-04321-x 31388884

[18] Falony G Joossens M Vieira-Silva S Wang J Darzi Y Faust K. Population-level analysis of gut microbiome variation. Science. (2016) 352:560–4. doi: 10.1126/science.aad3503 27126039

[19] Zhernakova A Kurilshikov A Bonder MJ Tigchelaar EF Schirmer M Vatanen T. Population-based metagenomics analysis reveals markers for gut microbiome composition and diversity. Science. (2016) 352:565–9. doi: 10.1126/science.aad3369, PMID: 27126040 PMC5240844

[20] Weersma RK Zhernakova A Fu J. Interaction between drugs and the gut microbiome. Gut. (2020) 69:1510–9. doi: 10.1136/gutjnl-2019-320204, PMID: 32409589 PMC7398478

[21] Spor A Koren O Ley R. Unravelling the effects of the environment and host genotype on the gut microbiome. Nat Rev Microbiol. (2011) 9:279–90. doi: 10.1038/nrmicro2540, PMID: 21407244

[22] Asnicar F Berry SE Valdes AM Nguyen LH Piccinno G Drew DA. Microbiome connections with host metabolism and habitual diet from 1,098 deeply phenotyped individuals. Nat Med. (2021) 27:321–32. doi: 10.1038/s41591-020-01183-8, PMID: 33432175 PMC8353542

[23] Goodrich JK Waters JL Poole AC Sutter JL Koren O Blekhman R. Human genetics shape the gut microbiome. Cells. (2014) 159:789–99. doi: 10.1016/j.cell.2014.09.053, PMID: 25417156 PMC4255478

[24] Shi N Li N Duan X Niu H. Interaction between the gut microbiome and mucosal immune system. Mil Med Res. (2017) 4:14. doi: 10.1186/s40779-017-0122-9, PMID: 28465831 PMC5408367

[25] Garrett-Bakelman F Darshi M Green S Gur R Lin L Macias B. The NASA twins study: a multidimensional analysis of a year-long human spaceflight. Science. (2019) 364:144. doi: 10.1126/science.aau8650 PMC7580864 30975860

[26] Chevalier C Stojanović O Colin DJ Suarez-Zamorano N Tarallo V Veyrat-Durebex C. Gut microbiota orchestrates energy homeostasis during cold. Cells. (2015) 163:1360–74. doi: 10.1016/j.cell.2015.11.004, PMID: 26638070

[27] Lian P Braber S Garssen J Wichers HJ Folkerts G Fink-Gremmels J. Beyond heat stress: intestinal integrity disruption and mechanism-based intervention strategies. Nutrients. (2020) 12:734. doi: 10.3390/nu12030734 32168808 PMC7146479

[28] Li L Zhao X. Comparative analyses of fecal microbiota in Tibetan and Chinese Han living at low or high altitude by barcoded 454 pyrosequencing. Sci Rep. (2015) 5:14682. doi: 10.1038/srep14682, PMID: 26443005 PMC4595765

[29] Mazel F. Living the high life: could gut microbiota matter for adaptation to high altitude? Mol Ecol. (2019) 28:2119–21. doi: 10.1111/mec.15093, PMID: 31127960

[30] Li K Peng W Zhou Y Ren Y Zhao J Fu X. Host genetic and environmental factors shape the composition and function of gut microbiota in populations living at high altitude. Biomed Res Int. (2020) 2020:1482109–10. doi: 10.1155/2020/1482109, PMID: 32190648 PMC7071804

[31] Casero D Gill K Sridharan V Koturbash I Nelson G Hauer-Jensen M. Space-type radiation induces multimodal responses in the mouse gut microbiome and metabolome. Microbiome. (2017) 5:105:105. doi: 10.1186/s40168-017-0325-z, PMID: 28821301 PMC5563039

[32] Mclaughlin MM Dacquisto MP Jacobus DP Horowitz RE. Effects of the germfree STATE on responses of mice to WHOLE-body irradiation. Radiat Res. (1964) 23:333–49. doi: 10.2307/3571614 14229117

[33] Packey CD Ciorba MA. Microbial influences on the small intestinal response to radiation injury. Curr Opin Gastroenterol. (2010) 26:88–94. doi: 10.1097/MOG.0b013e3283361927, PMID: 20040865 PMC4063200

[34] Wang J Han C Lu Z Ge P Cui Y Zhao D. Simulated microgravity suppresses MAPK pathway-mediated innate immune response to bacterial infection and induces gut microbiota dysbiosis. FASEB J. (2020) 34:14631–44. doi: 10.1096/fj.202001428R, PMID: 32918764

[35] Jiang P Green SJ Chlipala GE Turek FW Vitaterna MH. Reproducible changes in the gut microbiome suggest a shift in microbial and host metabolism during spaceflight. Microbiome. (2019) 7:113. doi: 10.1186/s40168-019-0724-4, PMID: 31399081 PMC6689164

[36] Ritchie LE Taddeo SS Weeks BR Lima F Bloomfield SA Azcarate-Peril MA. Space environmental factor impacts upon murine Colon microbiota and mucosal homeostasis. PLoS One. (2015) 10:e0125792. doi: 10.1371/journal.pone.0125792, PMID: 26083373 PMC4470690

[37] Voorhies A Ott C Mehta S Pierson D Crucian B Feiveson A. Study of the impact of long-duration space missions at the international Space Station on the astronaut microbiome. Sci Rep. (2019) 9:9911. doi: 10.1038/s41598-019-46303-8, PMID: 31289321 PMC6616552

[38] Saei A Barzegari A. The microbiome: the forgotten organ of the astronaut’s body - probiotics beyond terrestrial limits. Future Microbiol. (2012) 7:1037–46. doi: 10.2217/FMB.12.82, PMID: 22953705

[39] Turroni S Magnani M Kc P Lesnik P Vidal H Heer M. Gut microbiome and space travelers’ health: state of the art and possible pro/prebiotic strategies for long-term space missions. Front Physiol. (2020) 11:553929. doi: 10.3389/fphys.2020.553929, PMID: 33013480 PMC7505921

[40] Liu Z Luo G Du R Sun W Li J Lan H. Effects of spaceflight on the composition and function of the human gut microbiota. Gut Microbes. (2020) 11:807–19. doi: 10.1080/19490976.2019.1710091, PMID: 31924114 PMC7524348

[41] Wilson JW Ott CM Zu Bentrup KH Ramamurthy R Quick L Porwollik S. Space flight alters bacterial gene expression and virulence and reveals a role for global regulator Hfq. Proc Natl Acad Sci U S A. (2007) 104:16299–304. doi: 10.1073/pnas.0707155104, PMID: 17901201 PMC2042201

[42] Siddiqui R Akbar N Khan N. Gut microbiome and human health under the space environment. J Appl Microbiol. (2021) 130:14–24. doi: 10.1111/jam.14789, PMID: 32692438

[43] Ilyin VK Kiryukhina NV. Disruption of the colonization resistance syndrome in humans in altered habitats and its prevention. Acta Nat. (2014) 6:10–8. doi: 10.32607/20758251-2014-6-2-10-18 PMC4115221 25093106

[44] Mardanov AV Babykin MM Beletsky AV Grigoriev AI Zinchenko VV Kadnikov VV. Metagenomic analysis of the dynamic changes in the gut microbiome of the participants of the MARS-500 experiment, simulating long term space flight. Acta Nat. (2013) 5:116–25. doi: 10.32607/20758251-2013-5-3-116-125, PMID: 24303207 PMC3848073

[45] Chen Z Wang Z Li D Zhu B Xia Y Wang G. The gut microbiota as a target to improve health conditions in a confined environment. Front Microbiol. (2022) 13:1067756. doi: 10.3389/fmicb.2022.1067756, PMID: 36601399 PMC9806127

[46] Li L Su Q Xie B Duan L Zhao W Hu D. Gut microbes in correlation with mood: case study in a closed experimental human life support system. Neurogastroenterol Motil. (2016) 28:1233–40. doi: 10.1111/nmo.12822, PMID: 27027909

[47] Green MJ Aylott JW Williams P Ghaemmaghami AM Williams PM. Immunity in space: prokaryote adaptations and immune response in microgravity. Life. (2021) 11:112. doi: 10.3390/life11020112, PMID: 33540536 PMC7912908

[48] Page MJ McKenzie JE Bossuyt PM Boutron I Hoffmann TC Mulrow CD. Statement: an updated guideline for reporting systematic reviews. BMJ. (2020) 372:n71. doi: 10.1136/bmj.n71, PMID: 33782057 PMC8005924

[49] Schardt C Adams MB Owens T Keitz S Fontelo P. Utilization of the PICO framework to improve searching PubMed for clinical questions. BMC Med Inform Decis Mak. (2007) 7:16. doi: 10.1186/1472-6947-7-16, PMID: 17573961 PMC1904193

[50] Oren A Garrity GM. Valid publication of the names of forty-two phyla of prokaryotes. Int J Syst Evol Microbiol. (2021) 71:10. doi: 10.1099/ijsem.0.005056, PMID: 34694987

[51] Sterne JA Hernán MA Reeves BC Savović J Berkman ND Viswanathan M. ROBINS-I: a tool for assessing risk of bias in non-randomised studies of interventions. BMJ. (2016) 355:i4919. doi: 10.1136/bmj.i4919, PMID: 27733354 PMC5062054

[52] Turroni S Rampelli S Biagi E Consolandi C Severgnini M Peano C. Temporal dynamics of the gut microbiota in people sharing a confined environment, a 520-day ground-based space simulation, MARS500. Microbiome. (2017) 5:39. doi: 10.1186/s40168-017-0256-8 28340597 PMC5366131

[53] Brereton NJB Pitre FE Gonzalez E. Reanalysis of the Mars500 experiment reveals common gut microbiome alterations in astronauts induced by long-duration confinement. Comput Struct Biotechnol J. (2021) 19:2223–35. doi: 10.1016/j.csbj.2021.03.040, PMID: 33995915 PMC8099722

[54] Hao Z Li L Fu Y Liu H. The influence of bioregenerative life-support system dietary structure and lifestyle on the gut microbiota: a 105-day ground-based space simulation in lunar palace 1. Environ Microbiol. (2018) 20:3643–56. doi: 10.1111/1462-2920.14358, PMID: 30003647

[55] Meng C Wang W Hao Z Liu H. Investigation on the influence of isolated environment on human psychological and physiological health. Sci Total Environ. (2020) 716:136972. doi: 10.1016/j.scitotenv.2020.136972, PMID: 32036130

[56] Rerberg MS Manukovskiĭ NS Somova LA. Intestinal autoflora of the test subjects in a 6-month biological engineering experiment. Kosm Biol Aviakosm Med. (1977) 11:57–9. PMID: 15161

[57] Shilov VM Lizko NN Borisova OK Prokhorov VY. Changes in the microflora of man during long-term confinement. Life Sci Space Res. (1971) 9:43–49. 11942343

[58] Cordaro JT Sellers WM Ball RJ Schmidt JP. Study of man during a 56-day exposure to an oxygen-helium atmosphere at 258 mm. Hg total pressure X Enteric microbial flora. Aerosp Med. (1966) 37:594–6. 4381406

[59] Gall LS Riely PE. Effect of diet and atmosphere on intestinal and skin flora. I. Experimental data. NASA CR-661. NASA Contract Rep NASA CR. (1967) 1:1–215. 4381680

[60] Chen J Wang Q Hao Z Li Z Sahu S Liu H. Relationship between the gut microbiome and energy/nutrient intake in a confined bioregenerative life support system. Appl Environ Microbiol. (2020) 86:e02465. doi: 10.1128/AEM.02465-19, PMID: 31811045 PMC6997737

[61] Lizko N Shilov V Syrykh G Legenkov V. Composition on the intestinal microflora of cosmonauts before and after space missions. Kosm Biol Aviakosmicheskaya Med. (1979) 13:9–13. 388069

[62] Jin J Touyama M Yamada S Yamazaki T Benno Y. Alteration of a human intestinal microbiota under extreme life environment in the Antarctica. Biol Pharm Bull. (2014) 37:1899–906. doi: 10.1248/bpb.b14-00397, PMID: 25451839

[63] Zhang J Zhao J Jin H Lv R Shi H De G. Probiotics maintain the intestinal microbiome homeostasis of the sailors during a long sea voyage. Gut Microbes. (2020) 11:930–43. doi: 10.1080/19490976.2020.1722054, PMID: 32079472 PMC7524324

[64] Dong H Chen P Yu Y Zang P Wei Z. Simulated manned Mars exploration: effects of dietary and diurnal cycle variations on the gut microbiome of crew members in a controlled ecological life support system. PeerJ. (2019) 7:e7762. doi: 10.7717/peerj.7762, PMID: 31579622 PMC6766369

[65] Chen P Yu Y Tan C Liu H Wu F Li H. Human metabolic responses to microgravity simulated in a 45-day 6 degrees head-down tilt bed rest (HDBR) experiment. Anal Methods. (2016) 8:4334–44. doi: 10.1039/c6ay00644b

[66] Siddiqui R Qaisar R Goswami N Khan N Elmoselhi A. Effect of microgravity environment on gut microbiome and angiogenesis. Life (Basel). (2021) 11:1008. doi: 10.3390/life11101008, PMID: 34685381 PMC8541308

[67] Voorhies AA Lorenzi HA. The challenge of maintaining a healthy microbiome during long-duration space missions. Front Astron Space Sci. (2016) 3:23. doi: 10.3389/fspas.2016.00023

[68] Douglas GL DeKerlegand D Dlouhy H Dumont-Leblond N Fields E Heer M. Impact of diet on human nutrition, immune response, gut microbiome, and cognition in an isolated and confined mission environment. Sci Rep. (2022) 12:20847. doi: 10.1038/s41598-022-21927-5, PMID: 36522361 PMC9755260

[69] Vasiliev D. The role of biodiversity in ecosystem resilience. IOP Conf Ser: Earth Environ Sci. (2022) 1072:012012. doi: 10.1088/1755-1315/1072/1/012012

[70] Srivastava AK Rohil V Bhushan B Eslavath MR Gupta H Chanda S. Probiotics maintain the gut microbiome homeostasis during Indian Antarctic expedition by ship. Sci Rep. (2021) 11:18793. doi: 10.1038/s41598-021-97890-4, PMID: 34552104 PMC8458292

[71] Turnbaugh PJ Ridaura VK Faith JJ Rey FE Knight R Gordon JI. The effect of diet on the human gut microbiome: a metagenomic analysis in humanized Gnotobiotic mice. Sci Transl Med. (2009) 1:6ra14. doi: 10.1126/scitranslmed.3000322, PMID: 20368178 PMC2894525

[72] Zeng H. Mechanisms linking dietary fiber, gut microbiota and colon cancer prevention. World J Gastrointest Oncol. (2014) 6:41–51. doi: 10.4251/wjgo.v6.i2.41, PMID: 24567795 PMC3926973

[73] Tojo R. Intestinal microbiota in health and disease: role of bifidobacteria in gut homeostasis. World J Gastroenterol. (2014) 20:15163–76. doi: 10.3748/wjg.v20.i41.15163, PMID: 25386066 PMC4223251

[74] Boursier J Mueller O Barret M Machado M Fizanne L Araujo-Perez F. The severity of nonalcoholic fatty liver disease is associated with gut dysbiosis and shift in the metabolic function of the gut microbiota. Hepatology. (2016) 63:764–75. doi: 10.1002/hep.28356, PMID: 26600078 PMC4975935

[75] Lagier J-C Armougom F Million M Hugon P Pagnier I Robert C. Microbial culturomics: paradigm shift in the human gut microbiome study. Clin Microbiol Infect. (2012) 18:1185–93. doi: 10.1111/1469-0691.12023, PMID: 23033984

[76] Ji B Nielsen J. From next-generation sequencing to systematic modeling of the gut microbiome. Front Genet. (2015) 6:219. doi: 10.3389/fgene.2015.00219, PMID: 26157455 PMC4477173

[77] Relman DA. The human microbiome: ecosystem resilience and health. Nutr Rev. (2012) 70:S2–9. doi: 10.1111/j.1753-4887.2012.00489.x, PMID: 22861804 PMC3422777

[78] Dethlefsen L Relman DA. Incomplete recovery and individualized responses of the human distal gut microbiota to repeated antibiotic perturbation. Proc Natl Acad Sci U S A. (2011) 108:4554–61. doi: 10.1073/pnas.1000087107, PMID: 20847294 PMC3063582

[79] Van de Guchte M Burz SD Cadiou J Wu J Mondot S Blottière HM. Alternative stable states in the intestinal ecosystem: proof of concept in a rat model and a perspective of therapeutic implications. Microbiome. (2020) 8:153. doi: 10.1186/s40168-020-00933-7, PMID: 33158453 PMC7646066

