# Supplementary material for: Effects of isolation and confinement on gastrointestinal microbiota–a systematic review
Source: Front Nutr. 2023 Jul 10;10:1214016. doi: 10.3389/fnut.2023.1214016 (PMC10364611; doi:10.3389/fnut.2023.1214016)
Supplement: Supplementary file 1 [file Table_1.pdf]

Supplementary Material

Effects of isolation and confinement on gastrointestinal microbiota - a systematic review

Bea Klos[1], Christina Steinbach[1], Jasmin Ketel[1], Claude Lambert[2,3], John Penders[4,5], Joël Doré[6], Paul Enck[1], Isabelle Mack[1]*

[1]University Hospital Tübingen, Department of Psychosomatic Medicine and Psychotherapy, Tübingen, Germany

[2]CIRI – Immunology Lab University Hospital, Saint-Etienne, France

[3]LCOMS/ENOSIS Université de Lorraine, Metz, France

[4]CAPHRI Care and Public Health Research Institute, Department of Medical Microbiology, Infectious Diseases and Infection Prevention, Maastricht University Medical Center+, Maastricht, The Netherlands

[5]School of Nutrition and Translational Research in Metabolism, Department of Medical Microbiology, Infectious Diseases and Infection Prevention, Maastricht University Medical Center+, Maastricht, The Netherlands

[6]UMR Micalis Institut, INRA, Paris-Saclay University, Jouy-En-Josas, France.

*** Correspondence:** Dr. Isabelle Mack
E-Mail: isabelle.mack@uni-tuebingen.de

Keywords: isolation, confinement, human, microbiota, gut, gastrointestinal

1 Supplementary Tables

Table S1: Study outcomes of microbiota analysis by cultivation-based methods, in-mission (orange), post-mission (green) and pre-post-comparison (blue), outcomes are split according to corresponding isolation habitats

Study ID	Author (Year)	Subject	*Lactobacillus spp.*	*Bifidobacterium spp.*	*Escherichia spp.*	Other
**Subgroup 1: Isolation caused by space missions**						
**1**	*Lizko (1979)*	Sojus 17/1	↔ / ↓	N.R.	N.R.	N.R.
		Sojus 17/2	↔ / ↔ / ↔	↔ / ↔ / ↔	↔ / ↔ / ↔	↔ / ↔ / ↔
		Sojus 18/1	↔ / ↑ / ↓	↔ / ↑ / ↓	↔ / ↑ / ↓	Spore-forming bacteria ↔ / ↑ / ↓<br>*Proteus spp.* ↔ / ↔ / ↔
		Sojus 18/2	↔ / ↑ / ↓	↔ / ↑ / ↓	↔ / ↑ / ↓	Spore-forming bacteria ↔ / ↑ / ↓<br>*Proteus spp.* ↓
**Subgroup 2: Isolation caused by spaceflight- or gravity simulators**						
**2**	*Chen (2016)*	∑	↓/ ↓ / ↑	↓/ ↓ / ↑	N.R.	N.R.
**3**	*Cordaro (1966)*	∑	↔ / ↔ / ↔	N.R.	N.R.	*Bacteroides spp.* ↔ / ↔ / ↔<br>*Enterococci spp.* ↔ / ↓ / ↑<br>Coliforms ↔ / ↔ / ↔
**4**	*Gall (1964)*	1	↔	N.R.	↓ / ↑	*Klebsiella spp.* ↔<br>*Citrobacter spp.* ↓<br>*Shigella Boydii* ↓<br>*Corynebacteria spp.* ↑
		2	↔	N.R.	↔	*Corynebacteria spp.* ↑
		3	↔	N.R.	↔	*Klebsiella spp.* ↑<br>*Corynebacteria spp.* ↑
		4	↔	N.R.	↑	*Klebsiella spp.* ↑<br>*Corynebacteria spp.* ↑
**5**	*Rerberg (1967)*	∑	↓	↓	N.R.	*Bacteroides spp.* ↔<br>*Clostridium perfringens* ↑
**6**	*Shilov (1971)*	∑	↓ / ↓ / ↑	↓ / ↓ / ↑	N.R.	*Clostridium perfringens* ↔
			↓ / ↓ / ↔	↓ / ↓ / ↔	N.R.	*Clostridium perfringens* ↑
			↔	↔	N.R.	*Clostridium perfringens* ↑

Notes: The arrows represent the direction of the microbial abundance shift. ↓: abundance reduction, ↑: abundance increase, ↔ no change in abundance detected, the colors correspond to the sampling times (blue: change pre/post mission, orange: change pre/during mission, green: change during/post- mission, black: change without exact definition of the sampling time). *Abbreviations*: N.R.: not reported; spp.: multiple species of a genus.
