# Supplementary material for: Effects of isolation and confinement on gastrointestinal microbiota–a systematic review
Source: Front Nutr. 2023 Jul 10;10:1214016. doi: 10.3389/fnut.2023.1214016 (PMC10364611; doi:10.3389/fnut.2023.1214016)
Supplement: Supplementary file 2 [file Table_2.pdf]

Supplementary Material

Effects of isolation and confinement on gastrointestinal microbiota - a systematic review

Bea Klos[1], Christina Steinbach[1], Jasmin Ketel[1], Claude Lambert[2,3], John Penders[4,5], Joël Doré[6], Paul Enck[1], Isabelle Mack[1]*

[1]University Hospital Tübingen, Department of Psychosomatic Medicine and Psychotherapy, Tübingen, Germany

[2]CIRI – Immunology Lab University Hospital, Saint-Etienne, France

[3]LCOMS/ENOSIS Université de Lorraine, Metz, France

[4]CAPHRI Care and Public Health Research Institute, Department of Medical Microbiology, Infectious Diseases and Infection Prevention, Maastricht University Medical Center+, Maastricht, The Netherlands

[5]School of Nutrition and Translational Research in Metabolism, Department of Medical Microbiology, Infectious Diseases and Infection Prevention, Maastricht University Medical Center+, Maastricht, The Netherlands

[6]UMR Micalis Institut, INRA, Paris-Saclay University, Jouy-En-Josas, France.

*** Correspondence:** Dr. Isabelle Mack
E-Mail: isabelle.mack@uni-tuebingen.de

Keywords: isolation, confinement, human, microbiota, gut, gastrointestinal

1 Supplementary Tables

Table S2: Bacterial diversity and microbial abundance shifts in-mission (orange), post-mission (green) and pre-post-comparison (blue) analyzed by molecular-based approaches, outcomes are summarized on phylum level

Study			Microbial diversity shifts			Relative abundance shifts at phylum level					
**Study ID**	**Author (Year)**	**Subject**	**α-D: Richness**	**α-D: Biodiversity**	**Community structure**	**Bacillota**	**Bactero-idota**	**Actino-mycetota**	**Pseudo-monadota**	**Verruco-microbiota**	**Other**
**Subgroup 1: Isolation caused by space missions**											
**1**	*Garret-Bakelman (2019)*	∑	↔	↔	Dis.	Taxa shifts have not been assigned by name!					
**2**	*Liu (2020)*	A	↓ / ↑ / ↓		Dis.	↓ / ↑ / ↓	↑ / ↓ / ↑	↔ / ↑ / ↓	↓ / ↑ / ↓	↔ / ↑ / ↓	Fusobacteria ↑ / ↔ / ↑, Chlamydiae ↑ / ↓ / ↓, Tenericutes ↑ / ↓ / ↔, Aquificae ↔ / ↔ / ↔
		B	↔ / ↓ / ↑		Dis.	↑ / ↓ / ↑	↓ / ↑ / ↓	↑ / ↓ / ↑	↔	↓ / ↑ / ↓	Fusobacteria ↔ / ↔ / ↔, Chlamydiae ↑ / ↓ / ↑, Tenericutes ↓ / ↔ / ↓, Aquificae ↓ / ↓ / ↔
**3**	*Voorhies (2019)*	∑	↔ / ↑ / ↓	↔ / ↑ / ↓	Dis.	↔	↔	↔	↔	↔	↔
**Subgroup 2: Isolation caused by spaceflight- or gravity simulators**											
**4**	*Brereton (2021)*	∑	↔	↔	N.R.	↔	↔	↔	↔	↔	↔
**5**	Turroni *(2017)*	∑	N.R.	N.R.	Sim	↔	↔	↔	↔	↔	↔
**6**	*Mardanov (2013)*	A	N.R.	N.R.	N.R.	↔	↔	↔	↔	↔	↔
		B	N.R.	N.R.	N.R.	↔	↔	↔	↔	↑ / ↔ / ↑	Fusobacteria ↓ / ↓ / ↓
		C	N.R.	N.R.	N.R.	↔	↔	↔	↔	↓ / ↓ / ↔	↔
		D	N.R.	N.R.	N.R.	↔	↔	↑ / ↑ / ↓	↔	↔	↔
		E	N.R.	N.R.	N.R.	↔	↔	↔	↔	↔	↔
**7**	*Hao (2018)*	∑	↓ / ↑ / ↓		Sim.	↑ / ↓ / ↔	↓ / ↑ / ↔	↔	↔	↔	↔
**8**	*Li (2016)*	∑	N.R.	N.R.	N.R.	↔	↔	↔	↔	↔	↔
**9**	*Meng (2020)*	∑	N.R.	N.R.	N.R.	↔	↔	↔	↔	↔	↔
**10**	*Chen (2020)*	f/m	N.R.		Dis.	↔	↔	↔	↔	↔	↔
		F	↓ / ↓ / ↑			↔	↔	↔	↔	↔	↔
		M	↑ / ↑ / ↓			↔	↔	↔	↔	↔	↔
**11**	*Dong (2019)*	∑	↓	↓	N.R.	↓	↑	↔	↔	↔	Fusobacteria ↑
**Subgroup 3: Isolation in a natural, earth-bound habitat**											
**12**	*Jin (2014)*	∑	N.R.	N.R.	N.R.	↔	↔	↔	↔	↔	↔
**13**	*Zhang (2020)*	Plac.	↔	↔	Dis.	↔	↔	↔	↔	↔	↔
		Pro.	↔	↔	Sim.	↔	↔	↔	↔	↔	↔

Notes: The arrows represent the direction of the microbial abundance shift. ↓: abundance reduction, ↑: abundance increase, ↔ no change in abundance detected, the colors correspond to the sampling times (blue: change pre/post mission, orange: change pre/during mission, green: change during/post- mission, black: change without exact definition of the sampling time). The microbial community structure is described by the degree of similarity/dissimilarity of the microbial communities between different microbial habitats (in-mission samples), *: Trend to converge. The study subjects can be reported individually, in groups or summarized for all study participants (∑). *Abbreviations*: α-D.: alpha-Diversity; Dis.: dissimilar; f.: female; m.: male; N.R.: not reported; Plac.: Placebo group; Pro.: Group taking additional probiotics during the intervention; Sim.: similar.
