# Supplementary material for: Effects of isolation and confinement on gastrointestinal microbiota–a systematic review
Source: Front Nutr. 2023 Jul 10;10:1214016. doi: 10.3389/fnut.2023.1214016 (PMC10364611; doi:10.3389/fnut.2023.1214016)
Supplement: Supplementary file 3 [file Table_3.pdf]

Supplementary Material

Effects of isolation and confinement on gastrointestinal microbiota - a systematic review

Bea Klos[1], Christina Steinbach[1], Jasmin Ketel[1], Claude Lambert[2,3], John Penders[4,5], Joël Doré[6], Paul Enck[1], Isabelle Mack[1]*

[1]University Hospital Tübingen, Department of Psychosomatic Medicine and Psychotherapy, Tübingen, Germany

[2]CIRI – Immunology Lab University Hospital, Saint-Etienne, France

[3]LCOMS/ENOSIS Université de Lorraine, Metz, France

[4]CAPHRI Care and Public Health Research Institute, Department of Medical Microbiology, Infectious Diseases and Infection Prevention, Maastricht University Medical Center+, Maastricht, The Netherlands

[5]School of Nutrition and Translational Research in Metabolism, Department of Medical Microbiology, Infectious Diseases and Infection Prevention, Maastricht University Medical Center+, Maastricht, The Netherlands

[6]UMR Micalis Institut, INRA, Paris-Saclay University, Jouy-En-Josas, France.

*** Correspondence:** Dr. Isabelle Mack
E-Mail: isabelle.mack@uni-tuebingen.de

Keywords: isolation, confinement, human, microbiota, gut, gastrointestinal

1 Supplementary Tables

Table S3: Study outcomes for each subgroup and each sampling period at genus/species level, extended

			SUBGROUP 1: SPACE			SUBGROUP 2: SIMULATION			SUBGROUP 3: NATURAL, EARTH-BOUND		
ID	PHYLUM	GENUS	IN-MISSION	POST-MISSION	PRE-/POST-MISSION	IN-MISSION	POST-MISSION	PRE-/POST-MISSION	IN-MISSION	POST-MISSION	PRE-/POST-MISSION
3	Verrucomicrobia	*Akkermansia*	↓		**R**						
5	Verrucomicrobia	*Akkermansia sp.*					↓				
2A	Bacteroidetes	*Alistipes*	↑	↓	↔						
2B	Bacteroidetes	*Alistipes*	↑	↓	↓						
6	Bacteroidetes	*Alistipes*				↑	↔	**pR**			
11	Bacteroidetes	*Alistipes*				↓					
13Pla	Bacteroidetes	*Alistipes sp.*							↓		
13Pro	Bacteroidetes	*Alistipes sp.*							↓		
10	Firmicutes	*Anaerostipes sp.*				↓					
4	Firmicutes	*Anaerostipes sp.*				↓					
2A	Bacteroidetes	*Bacteroides*	↓	↑	↑						
2B	Bacteroidetes	*Bacteroides*	↑	↓	↓						
6	Bacteroidetes	*Bacteroides*				↓	↑	**pR**			
6	Bacteroidetes	*Bacteroides*				↓	↔	**pR**			
6	Bacteroidetes	*Bacteroides*				↔	↑	**pR**			
6	Bacteroidetes	*Bacteroides*				↓	↓	**pR**			
4	Bacteroidetes	*Bacteroides*				↑					
11	Bacteroidetes	*Bacteroides*				↓					
12A	Bacteroidetes	*Bacteroides*							↓	↑	**R**
5	Bacteroidetes	*Bacteroides sp.*				↑					
10	Bacteroidetes	*Bacteroides sp.*				↓					
10	Bacteroidetes	*Bacteroides sp.*				↓					
13Pla	Bacteroidetes	*Bacteroides sp.*							↓		
2A	Actinobacter	*Bifidobacterium*	↔	↔	↔						
2B	Actinobacter	*Bifidobacterium*	↓	↑	↑						
12A	Actinobacter	*Bifidobacterium*							↓	↑	**R**
12C	Actinobacter	*Bifidobacterium*							↓	↑	**R**
12E	Actinobacter	*Bifidobacterium*							↓	↑	**R**
12B	Actinobacter	*Bifidobacterium*							↑		
10	Actinobacter	*Bifidobacterium sp.*				↓					

13Pro	Actinobacter	*Bifidobacterium sp.*							↑		
13Pro	Actinobacter	*Bifidobacterium sp.*							↑		
2A	Firmicutes	*Blautia*	↑	↓	↔						
2B	Firmicutes	*Blautia*	↔	↑	↑						
7	Firmicutes	*Blautia*				↑	↓				
4	Firmicutes	*Blautia sp.*				↓					
11	Bacteroidetes	*Butyricimonas*				↓					
4	Firmicutes	*Christensenellaceae*				↑					
2A	Firmicutes	*Clostridium*	↑	↓	↓						
2B	Firmicutes	*Clostridium*	↔	↓	↓						
4	Firmicutes	*Clostridium sp.*				↓					
10	Firmicutes	*Clostridium sp.*				↓					
13Pro	Firmicutes	*Clostridium sp.*							↓		
12A	Firmicutes	*Clostridium subcluster IV*							↓		
12D	Firmicutes	*Clostridium subcluster IV*							↓		
12A	Firmicutes	*Clostridium subcluster XIVa*							↑		
12B	Firmicutes	*Clostridium subcluster XIVa*							↑		
12C	Firmicutes	*Clostridium subcluster XIVa*							↑		
12D	Firmicutes	*Clostridium subcluster XIVa*							↑		
12E	Firmicutes	*Clostridium subcluster XIVa*							↑		
12F	Firmicutes	*Clostridium subcluster XIVa*							↑		
12F	Firmicutes	*Clostridium subcluster XIVa*							↓	↓	
12A	Firmicutes	*Clostridium subcluster XVIII*							↑	↑	
12B	Firmicutes	*Clostridium subcluster XVIII*							↑	↑	
12C	Firmicutes	*Clostridium subcluster XVIII*							↑	↑	
12D	Firmicutes	*Clostridium subcluster XVIII*							↑	↑	
12E	Firmicutes	*Clostridium subcluster XVIII*							↑	↑	
12F	Firmicutes	*Clostridium subcluster XVIII*							↑	↑	
3	Firmicutes	*Coprococcus*			↓						
5	Firmicutes	*Coprococcus sp.*				↔					
6	Firmicutes	*Coprococcus sp.*						**pR**			
6	Firmicutes	*Dialister*				↔	↓	**pR**			
6	Firmicutes	*Dialister*				↔	↔	**pR**			

6	Firmicutes	*Dialister*				↔	↔	**pR**			
6	Firmicutes	*Dialister*				↔	↔	**pR**			
11	Firmicutes	*Dialister*				↓					
3	Firmicutes	*Dorea*	↓		**R**						
5	Firmicutes	*Dorea sp.*				↔					
10	Proteobacteria	*Enterobacter sp.*				↓					
12F	Firmicutes	*Enterobacteriales*							↓	↓	
2A	Proteobacteria	*Escherichia*	↓	↓	↓						
2B	Proteobacteria	*Escherichia*	↓	↑	↔						
10	Proteobacteria	*Escherichia sp.*				↓					
2A	Firmicutes	*Eubacterium*	↓	↓	↑						
2B	Firmicutes	*Eubacterium*	↑	↓	↔						
3	Firmicutes	*Eubacterium sp.*	↓		**R**						
2A	Firmicutes	*Faecalibacterium*	↔	↓	↓						
2B	Firmicutes	*Faecalibacterium*	↓	↓	↓						
3	Firmicutes	*Faecalibacterium*	↑		**R**						
7	Firmicutes	*Faecalibacterium*				↑	↓				
11	Firmicutes	*Faecalibacterium*				↑					
10	Firmicutes	*Faecalibacterium sp.*				↓					
4	Firmicutes	*Faecalibacterium sp.*				↓					
4	Firmicutes	*Faecalibacterium sp.*				↓					
4	Firmicutes	*Faecalibacterium sp.*				↓					
4	Firmicutes	*Faecalibacterium sp.*				↓					
5	Firmicutes	*Faecalibacterium sp.*				↓					
6	Firmicutes	*Faecalibacterium sp.*						**pR**			
6	Firmicutes	*Faecalibacterium sp.*						**pR**			
6	Firmicutes	*Faecalibacterium sp.*						**pR**			
13Pla	Firmicutes	*Flavonifractor*							↓		
13Pro	Firmicutes	*Flavonifractor sp.*							↓		
3	Firmicutes	*Fusicatenibacter*	↑		**R**						
4	Firmicutes	*Kineothrix*				↑					
13Pro	Proteobacteria	*Klebsiella sp.*							↓		
3	Firmicutes	*Lachnospira*	↑		**R**						
7	Firmicutes	*Lachnospira*				↑	↓				

11	Firmicutes	*Lachnospira*				↓					
4	Firmicutes	*Lachnospira*				↓					
3	Firmicutes	*Lachnospiraceae*	↑		**R**						
4	Firmicutes	*Lachnospiraceae*				↑					
10	Firmicutes	*Lachnospiraceae*				↑					
2	Firmicutes	*Lactobacillus*	↑	↓	↓						
2	Firmicutes	*Lactobacillus*	↓	↔	↓						
4	Firmicutes	*Lactobacillus sp.*				↓					
13Pla	Firmicutes	*Lactobacillus sp.*							↑		
13Pla	Firmicutes	*Lactococcus sp.*							↓		
3	Fusobacteria	*Leptotrichia*	↓		**R**						
6	Firmicutes	*Megamonas*				↑	↑	**pR**			
3	Firmicutes	*Megasphaera*	↓		**R**						
11	Bacteroidetes	*Parabacteroides*				↓					
3	Proteobacteria	*Parasutterella*	↑		**R**						
6	Firmicutes	*Phascolarctobacterium*				↔	↑	**pR**			
6	Firmicutes	*Phascolarctobacterium*				↓	↔	**pR**			
6	Firmicutes	*Phascolarctobacterium*				↔	↓	**pR**			
6	Firmicutes	*Phascolarctobacterium*				↔	↔	**pR**			
6	Firmicutes	*Phascolarctobacterium*				↔	↔	**pR**			
2A	Bacteroidetes	*Prevotella*	↑	↓	↔						
2B	Bacteroidetes	*Prevotella*	↑	↓	↓						
3	Bacteroidetes	*Prevotella*	↓		**R**						
6	Bacteroidetes	*Prevotella*				↓	↓	↑			
6	Bacteroidetes	*Prevotella*				↔	↑	↓			
7	Bacteroidetes	*Prevotella*				↓	↑				
11	Bacteroidetes	*Prevotella*				↑					
10	Bacteroidetes	*Prevotella sp.*				↑					
13Pla	Bacteroidetes	*Prevotella sp.*							↓		
13Pro	Bacteroidetes	*Prevotella sp.*							↓		
3	Firmicutes	*Pseudobutyrivibrio*	↓		**R**						
11	Firmicutes	*Pseudobutyrivibrio*				↑					
2A	Firmicutes	*Roseburia*	↑	↓	↓						
2B	Firmicutes	*Roseburia*	↑	↓	↓						

4	Firmicutes	*Roseburia*				↓					
4	Firmicutes	*Roseburia sp.*				↓					
13Pla	Firmicutes	*Roseburia sp.*							↓		
13Pro	Firmicutes	*Roseburia sp.*							↓		
3	Firmicutes	*Ruminiclostridium*	↑		**R**						
4	Firmicutes	*Ruminococcaceae sp.*				↑					
3	Firmicutes	*Ruminococcus*	↓		↓						
11	Firmicutes	*Ruminococcus*				↓					
4	Firmicutes	*Ruminococcus sp.*				↑					
4	Firmicutes	*Ruminococcus sp.*				↓					
3	Firmicutes	*Streptococcus*	↓		**R**						
4	Firmicutes	*Streptococcus sp.*				↑					
13Pla	Firmicutes	*Streptococcus sp.*							↓		
13Pro	Firmicutes	*Streptococcus sp.*							↓		
13Pla	Firmicutes	*Streptococcus sp.*							↑		
13Pla	Firmicutes	*Streptococcus sp.*							↑		
13Pla	Firmicutes	*Streptococcus sp.*							↑		
11	Firmicutes	*Subdoligranulum*				↑					
3	Firmicutes	*Veillonella*	↓		**R**						
10	Firmicutes	*Veillonella sp.*				↓					

Notes: Microbiota abundance shifts/ continuities identified at genus (+ species) level in 1) Subgroup 1: Isolation in Space, 2) Subgroup 2: Isolation in a space- or microgravity-simulation unit and 3) Subgroup 3: Natural, earth bound isolation either i) through mthe intervention compared to baseline (in-mission) or ii) after the isolation period compared to the intervention (post-mission) or iii) after the isolation compared to baseline (pre-/post-mission). The arrows represent the direction of the microbial shift. ↓: reduction, ↑: increase, ↔ no (significant) change detected, R Restored back to baseline level, pR: partly restored back to baseline.

Abbreviations: Plac.: Placebo group; Pro.: Group taking additional probiotics during the intervention.
