# Supplementary material for: Effects of isolation and confinement on gastrointestinal microbiota–a systematic review
Source: Front Nutr. 2023 Jul 10;10:1214016. doi: 10.3389/fnut.2023.1214016 (PMC10364611; doi:10.3389/fnut.2023.1214016)
Supplement: Supplementary file 4 [file Data_Sheet_1.pdf]

Supplementary Material

Effects of isolation and confinement on gastrointestinal microbiota - a systematic review

Bea Klos[1], Christina Steinbach[1], Jasmin Ketel[1], Claude Lambert[2,3], John Penders[4,5], Joël Doré[6], Paul Enck[1], Isabelle Mack[1]*

[1]University Hospital Tübingen, Department of Psychosomatic Medicine and Psychotherapy, Tübingen, Germany

[2]CIRI – Immunology Lab University Hospital, Saint-Etienne, France

[3]LCOMS/ENOSIS Université de Lorraine, Metz, France

[4]CAPHRI Care and Public Health Research Institute, Department of Medical Microbiology, Infectious Diseases and Infection Prevention, Maastricht University Medical Center+, Maastricht, The Netherlands

[5]School of Nutrition and Translational Research in Metabolism, Department of Medical Microbiology, Infectious Diseases and Infection Prevention, Maastricht University Medical Center+, Maastricht, The Netherlands

[6]UMR Micalis Institut, INRA, Paris-Saclay University, Jouy-En-Josas, France.

*** Correspondence:** Dr. Isabelle Mack
E-Mail: isabelle.mack@uni-tuebingen.de

Keywords: isolation, confinement, human, microbiota, gut, gastrointestinal

1 Supplementary Text

Text S1: DATABASE 1 - PUBMED:

((("Antarctic Regions"[MeSH Terms] OR "Antarct*"[Title/Abstract] OR "Arctic Regions"[MeSH Terms] OR "Arctic*"[Title/Abstract] OR "Sub-Antarctic*"[Title/Abstract] OR "Space Simulation"[MeSH Terms] OR "Ships"[MeSH Terms] OR "Moon"[MeSH Terms] OR "Mars"[MeSH Terms] OR "Spacecraft"[MeSH Terms] OR "Extraterrestrial Environment"[MeSH Terms] OR "Biosphere 2"[Title/Abstract] OR "Envihab"[Title/Abstract] OR "Mars-500"[Title/Abstract] OR "Mars-105"[Title/Abstract] OR "SFINCSS-99"[Title/Abstract] OR "Concordia Station"[Title/Abstract] OR "Zhongshan Station"[Title/Abstract] OR "Neumayer"[Title/Abstract] OR "Maitri Station"[Title/Abstract] OR "Great Wall Station"[Title/Abstract] OR "Davis Station"[Title/Abstract] OR "McMurdo Station"[Title/Abstract] OR "Palmer Station"[Title/Abstract] OR "South Pole"[Title/Abstract] OR "Arctowski"[Title/Abstract] OR "Vostok Station"[Title/Abstract]

OR "Syowa Station"[Title/Abstract] OR "HI-SEAS"[Title/Abstract] OR "polar institute*"[Title/Abstract] OR "AGBRESA"[Title/Abstract] OR "SIRIUS"[Title/Abstract] OR "Space Station"[Title/Abstract] OR "Apollo"[Title/Abstract] OR "Lunar Palace"[Title/Abstract] OR "submarine*"[Title/Abstract] OR "land based simulator*"[Title/Abstract] OR "Space condition*"[Title/Abstract] OR "long duration space exploration mission*"[Title/Abstract] OR "polar expedition*"[Title/Abstract] OR "Space Flight"[MeSH Terms] OR "Aerospace Medicine"[MeSH Terms] OR "Astronauts"[MeSH Terms] OR "space flight*"[Title/Abstract] OR "spaceflight*"[Title/Abstract] OR "Space Travel*"[Title/Abstract] OR "space explore*"[Title/Abstract] OR "Space Mission*"[Title/Abstract] OR "astronaut*"[Title/Abstract] OR "cosmonaut*"[Title/Abstract] OR "International Space Station"[Title/Abstract] OR "Mir Space Station"[Title/Abstract] OR "Long-duration spaceflight"[Title/Abstract]) OR (("Siberia"[MeSH Terms] OR "Extreme Environments"[MeSH Terms] OR "environment, controlled"[MeSH Terms] OR "Atlantic Ocean"[MeSH Terms] OR "Pacific Ocean"[MeSH Terms] OR "United States National Aeronautics and Space Administration"[MeSH Terms] OR "Seasons"[MeSH Terms] OR "Cold Climate"[MeSH Terms] OR "Cold Temperature"[MeSH Terms] OR "Darkness"[MeSH Terms] OR "Sunlight"[MeSH Terms] OR "Temperature"[MeSH Terms] OR "hypoxia/diet therapy"[MeSH Terms] OR "hypoxia/physiology"[MeSH Terms] OR "hypoxia/physiopathology"[MeSH Terms] OR "International Cooperation*"[MeSH Terms] OR "Workplace"[MeSH Terms] OR "Biomedical Research"[MeSH Terms] OR "Siberia"[Title/Abstract] OR "extreme environment*"[Title/Abstract] OR "extreme living condition*"[Title/Abstract] OR "Winterover"[Title/Abstract] OR "Winter-over"[Title/Abstract] OR "overwintering*"[Title/Abstract] OR "team member*"[Title/Abstract] OR "crew member*"[Title/Abstract] OR "research station*"[Title/Abstract] OR "simulation facility*"[Title/Abstract] OR "challenging habitat*"[Title/Abstract] OR "habitat research"[Title/Abstract] OR "human habitation*"[Title/Abstract] OR "Mars analog mission"[Title/Abstract] OR "Polar Research"[Title/Abstract] OR "Earth-based analogs"[Title/Abstract] OR "Ground analogs"[Title/Abstract] OR "NASA"[Title/Abstract] OR "Space Mission*"[Title/Abstract] OR "ISS"[Title/Abstract] OR "Euromir"[Title/Abstract] OR "Skylab"[Title/Abstract] OR "Space Administration"[Title/Abstract] OR "European Space Agency"[Title/Abstract] OR "ESA"[Title/Abstract] OR "season*"[Title/Abstract] OR "simulation condition*"[Title/Abstract] OR "Hypobaria"[Title/Abstract] OR "Hypoxia"[Title/Abstract] OR "high altitude"[Title/Abstract] OR "very low temperature"[Title/Abstract] OR "Extreme Medicine"[Title/Abstract] OR "Physiological System"[Title/Abstract] OR "Physiological Challenge"[Title/Abstract] OR "biomedical research project*"[Title/Abstract]) AND ("Confined Spaces"[MeSH Terms] OR "Isolated Habitat*"[Title/Abstract] OR "Ecological Systems, Closed"[MeSH Terms] OR "Breadboard Project"[Title/Abstract] OR "Regenerative Life Support System*" [Title/Abstract] OR "Bioregenerative life-support*"[Title/Abstract] OR "Sealed Cabin Ecology"[Title/Abstract] OR "CELSS"[Title/Abstract] OR (("Bed Rest"[Mesh] OR "Bed Rest*"[Title/Abstract]) AND ("Longterm"[Title/Abstract] OR "Long Term"[Title/Abstract])) OR "Ground-based stud*"[Title/Abstract] OR "closed-chamber stud*"[Title/Abstract] OR "ICE-environment*"[Title/Abstract] OR "ICE-research*"[Title/Abstract] OR "ICE-condition*"[Title/Abstract] OR "ICE-stud*"[Title/Abstract] OR "Isolated environment*"[Title/Abstract] OR "Confinement*"[Title/Abstract] OR "Confined environment*"[Title/Abstract] OR "Confinement condition*"[Title/Abstract] OR "Confined condition*"[Title/Abstract] OR "Indoor Workplace*"[Title/Abstract] OR "Isolation at sea"[Title/Abstract] OR "Closed environment*"[Title/Abstract] OR "Closed Ecological System*"[Title/Abstract] OR "Semiclosed environment*"[Title/Abstract] OR "Isolation period*"[Title/Abstract] OR "Limited antigen*"[Title/Abstract] OR "Limited

antimicrobial*"[Title/Abstract] OR "Human adapti*"[Title/Abstract] OR "Winter-over syndrome"[Title/Abstract] OR "Polar T3 Syndrome"[Title/Abstract] OR "subsyndromal seasonal affective disorder*"[Title/Abstract]))) AND ("Gastrointestinal Microbiome"[MeSH Terms] OR (("Microbiome*"[Title/Abstract] OR "Microflora*"[Title/Abstract] OR "Microbiota*"[Title/Abstract] OR "Flora"[Title/Abstract]) AND ("Gut"[Title/Abstract] OR "Gastrointestinal"[Title/Abstract] OR "Intestinal"[Title/Abstract])))) NOT ("Animals"[MeSH Terms] NOT ("Humans"[MeSH Terms] OR Human*[Title/Abstract]))

DATABASE 2 - WEB OF SCIENCE:

1 TS=(Antarcti* OR Arctic* OR "Space Simulation*" OR Ships OR Moon OR Mars OR Spacecraft OR "Extraterrestrial Environments" OR "Biosphere 2" OR Envihab OR "SFINCSS-99" OR "Concordia Station" OR "Zhongshan Station" OR "Neumayer" OR "Maitri Station" OR "Great Wall Station" OR "Davis Station" OR "McMurdo Station" OR "Palmer Station" OR "South Pole" OR Arctowski OR "Vostok Station" OR "Syowa Station" OR "HI-SEAS" OR "Polar institute*" OR AGBRESA OR SIRIUS OR "Space Station*" OR Apollo OR "Lunar Palace" OR Submarine* OR "Land-based simulator*" OR "Space condition*" OR "Long-duration space exploration mission*" OR "Polar expedition*"))

#2 TS=("Space Flight*" OR Spaceflight* OR "Aerospace Medicine" OR "Space Travel*" OR "Space Explore*" OR Astronaut* OR Cosmonaut* OR "International Space Station" OR "Mir Space Station" OR ("Long-duration" NEAR/2 spaceflight*))

#3 TS=(Siberia* OR "Extreme Environment*" OR "Atlantic Ocean" OR "Pacific Ocean" OR "United States National Aeronautics and Space Administration" OR "Biomedical Research" OR "Extreme living condition*" OR Winterover OR Winter-over OR Overwintering* OR "Research Station*" OR "Simulation Facility*" OR "Challenging habitat*" OR (habitat NEAR/2 research) OR "Human Habitation*" OR (Polar NEAR/2 Research) OR "Earth-based analogs" OR "Ground analogs" OR NASA OR "European Space Agency" OR ESA OR Euromir OR Skylab OR (Space NEAR/2 Mission) OR Season* OR "Simulation condition*" OR Hypobaria OR Hypoxia OR "high altitude" OR "very low temperature*" OR "Extreme Medicine" OR (Physiological NEAR/2 (System* OR Challenge*)) OR (Biomedical NEAR/2 "research project*"))

#4 TS=((Confined NEAR/2 (Space* OR environment* OR condition*)) OR "Closed Ecological System*" OR "Semiclosed environment*" OR "Ground-based study" OR "Ground-based studies" OR "closed-chamber study" OR "closed-chamber studies" OR "ICE-environment*" OR "ICE-research" OR "ICE-condition*" OR "ICE-studies" OR "ICE-study" OR (Isolated NEAR/2 (environment* OR habitat*)) OR Confinement* OR (Isolation NEAR/5 sea) OR "Closed environment*" OR "isolation period*" OR "Human adaption" OR "Winter-over syndrome" OR "Polar T3 Syndrome" OR "Subsyndromal seasonal affective disorder*" OR "Breadboard Project" OR "Regenerative Life Support System*" OR "Bioregenerative life-support*" OR "Sealed Cabin Ecology" OR CELSS OR ("Bed Rest" NEAR/5 (Longterm OR "Long Term")) "Limited antigen*" OR "Limited antimicrobial*")

#5 TS=(Microbiome* OR Microflora* OR Microbiota* OR Flora) NEAR/5 (Gut OR Gastrointestinal* OR Intestinal* OR Intestine*))

#6 TS=(Animal* NOT Human*)

#7 #3 AND #4

#8 #1 OR #2 OR #7

#9 #8 AND #5 NOT #6

DATABASE 3 – COCHRANE LIBRARY:

#1 (Antarcti* OR Arctic* OR (Space near/3 Simulation*) OR Ships OR Moon OR Mars OR Spacecraft OR (Extraterrestrial near/2 Environment*) OR "Biosphere 2" OR Envihab OR "SFINCSS-99" OR "Concordia Station" OR "Zhongshan Station" OR "Neumayer" OR "Maitri Station" OR "Great Wall Station" OR "Davis Station" OR "McMurdo Station" OR "Palmer Station" OR "South Pole" OR Arctowski OR "Vostok Station" OR "Syowa Station" OR "HI-SEAS" OR "Polar institute*" OR AGBRESA OR SIRIUS OR "Space Station*" OR Apollo OR "Lunar Palace" OR Submarine* OR "Land-based simulator*" OR "Space condition*" OR "Long-duration space exploration mission*" OR (Polar near/2 expedition*)):ti,ab,kw

#2 ((Space near/2 (Flight* OR Travel* OR Explor*)) OR Spaceflight* OR (Aerospace near/2 Medicine) OR Astronaut* OR Cosmonaut* OR "International Space Station" OR (Mir near/3 Space Station) OR ("Long-duration" near/2 spaceflight)):ti,ab,kw

#3 (Siberia* OR (Extreme near/2 Environment*) OR "Atlantic Ocean" OR "Pacific Ocean" OR "United States National Aeronautics and Space Administration" OR (Biomedical near/3 Research) OR (Extreme near/3 "living condition*") OR Winterover OR Winter-over OR Overwintering* OR (Research near/2 Station*) OR (Simulation near/2 (Facilit* OR condition*)) OR "Challenging habitat*" OR (habitat near/2 research) OR "Human Habitation*" OR (Polar near/2 Research) OR "Earth-based analogs" OR "Ground analogs" OR NASA OR "European Space Agency" OR ESA OR Euromir OR Skylab OR (Space near/2 Mission) OR Season* OR Hypobaria OR Hypoxia OR "high altitude" OR "very low temperature*" OR "Extreme Medicine" OR (Physiological near/2 (System* OR Challenge*)) OR (Biomedical near/2 "research project*")):ti,ab,kw

#4 ((Confined near/2 (Space* OR environment* OR condition*)) OR (Closed near/3 "Ecological System*") OR (Semiclosed near/2 environment*) OR "Ground-based study" OR "Ground-based studies" OR "closed-chamber study" OR "closed-chamber studies" OR "ICE-environment*" OR "ICE-research" OR "ICE-condition*" OR "ICE-studies" OR "ICE-study" OR ((Isolated OR Closed) near/2 Environment*) OR Confinement* OR (Isolation near/5 Sea) OR (Isolation near/2 period*) OR "Human adaption" OR "Winter-over syndrome" OR "Polar T3 Syndrome" OR "Subsyndromal seasonal affective disorder*" OR "Breadboard Project" OR "Regenerative Life Support System*" OR (Bioregenerative near/2 life-support*) OR "Sealed Cabin Ecology" OR CELSS OR ("Bed Rest" near/5 (Longterm OR "Long Term")) "Limited antigen*" OR "Limited antimicrobial*"):ti,ab,kw

#5 ((Microbiome* OR Microflora* OR Microbiota* OR Flora) near/5 (Gut OR Gastrointestinal* OR Intestinal* OR Intestine*)):ti,ab,kw

#6 ([mh "Animals"] OR animal*:ti,ab,kw) NOT ([mh "Humans"] OR human*:ti,ab,kw)

#7 #3 AND #4

#8 #1 OR #2 OR #7

#9 #8 AND #5

#10 #9 NOT #6
